# Potential of low-enthalpy geothermal energy to degrade organic contaminants of emerging concern in urban groundwater

**DOI:** 10.1038/s41598-023-29701-x

**Published:** 2023-02-14

**Authors:** Estanislao Pujades, Anna Jurado, Laura Scheiber, Marc Teixidó, Rotman A. Criollo Manjarrez, Enric Vázquez-Suñé, Victor Vilarrasa

**Affiliations:** 1 Department of Geosciences, Institute of Environmental Assessment and Water Research (IDAEA), Severo Ochoa Excellence Center of the Spanish Council for Scientific Research (CSIC), Jordi Girona 18–26, 08034 Barcelona, Spain; 2 Global Change Research Group (GCRG), IMEDEA, CSIC-UIB, Miquel Marqués 21, 07190 Esporles, Spain

**Keywords:** Environmental sciences, Hydrology, Energy science and technology

## Abstract

Low-enthalpy geothermal energy (LEGE) is a carbon-free and renewable source to provide cooling and heating to infrastructures (e.g. buildings) by exchanging their temperature with that of the ground. The exchange of temperature modifies the groundwater temperature around LEGE installations, which may contribute to enhancing the capacity of aquifers to degrade organic contaminants of emerging concern (OCECs), whose presence is significantly increasing in urban aquifers. Here, we investigate the impact of LEGE on OCECs and their bioremediation potential through numerical modelling of synthetic and real-based cases. Simulation results demonstrate that: (i) LEGE facilities have the potential to noticeably modify the concentrations of OCECs; and (ii) the final impact depends on the design of the facility. This study suggests that optimized LEGE facility designs could contribute to the degradation of OCECs present in urban aquifers, thus improving groundwater quality and increasing its availability in urban areas.

## Introduction

As a result of climate change and growing global population, pressure on water resources is constantly increasing. The latter is especially dramatic in urban areas, which are expected to amass 70% of the world population by 2050^1^. In this context, it is of paramount importance to increase the availability of water resources by improving and preserving their quality. Urban aquifers have the potential to be used to cover the growing demand of tap water in urban areas and as a strategic resource during drought periods. However, urban aquifers are commonly contaminated by a vast array of pollutants, such as organic contaminants of emerging concern (OCECs) and their transformation products (TPs). OCECs and TPs, which are profuse in surface water bodies because wastewater treatment plants cannot remove them completely, reach urban aquifers through different recharge sources (e.g. artificial recharge, water leakage from sewer and septic systems, seepage from rivers, etc.).

OCECs comprise natural (e.g. hormones) and anthropogenic substances (e.g. surfactants, personal care products, pharmaceuticals, illicit drugs, pesticides, and corrosion inhibitor additives, among others) and are frequently reported in aquifers at low concentrations (from ng L^−1^ to µg L^−1^)^2,3^. However, even at these low concentrations, OCECs pose human health and ecological risks. OCECs may affect the endocrine system of organisms, induce microbiological resistance and be accumulated in ecosystems^4^. In general, their effects on soil, plants, animals and human health are largely unknown^5^, but are expected to be harmful.

Aquifers have the capacity to attenuate concentrations of OCECs, as demonstrated by the lower concentrations found in aquifers compared with those observed at their related rivers^6^. OCECs in aquifers are mainly degraded by microbial activity, since adsorption processes only retard their transport^7^. Herein, we refer to degradation as full elimination and transformation due to (a)biotic processes. Microbial degradation of OCECs is a redox-dependent process^8–13^ that could be enhanced by increasing the temperature^14,15^. Groundwater temperature is practically constant during the year^16^ (with the exception of aquifer areas very close to surface water bodies). Therefore, it is expected that, in most cases, temperature variations are not meaningful in the processes degrading OCECs.

However, anthropogenic activities can significantly modify groundwater temperature, especially in urban aquifers, where the subsurface is used for different purposes^17–19^. One of these activities consist in the use of the subsurface for cooling and heating of buildings and other infrastructures by means of low-enthalpy geothermal energy (LEGE), which does not emit greenhouse gases and is a renewable energy with great potential to mitigate climate change. LEGE can be defined as the energy stored in the first 400-m depth from the ground^20,21^, where temperatures typically remain below 30 °C and, thus, it is also known as low-temperature geothermal energy or shallow-geothermal energy^22^. The use of LEGE in urban areas has significantly increased during the last decade^23^, and today, the market growth rate shows a steady trend of 9%^24^. LEGE is based on groundwater temperature being nearly constant over time^25,26^, just barely affected by the seasonal variations of atmospheric temperature. Consequently, LEGE uses groundwater for heating and/or cooling by exchanging the groundwater heat with that of buildings through heat exchangers^27^. In summer, groundwater has a lower temperature than the atmospheric one, thus, groundwater can be used for cooling by transferring the heat of the building to groundwater, which produces the rise of aquifer temperature. In winter, the system is reversed and groundwater is used to heat the building by transferring the groundwater heat to the building, which reduces the aquifer temperature^28,29^. Thus, the use of groundwater for cooling and heating induces variations of aquifer temperature^30,31^. Our hypothesis is that temperature changes induced by LEGE systems can significantly modify the degradation rates of OCECs, purifying groundwater.

The impact of LEGE systems on organic pollutants has only been investigated for the case of chlorinated organic compounds (COCs) and in very few sites. A pioneering LEGE system was designed with remediation purposes in Eindhoven, the Netherlands, in a former industrial site contaminated with COCs^32^. Monitoring revealed that dechlorination capacity was improved, at least, at one of the monitoring wells^33^. Other successful studies on removing COCs from the subsurface, by taking advantage of LEGE, have been performed at the Welgelegen (the Netherlands) and the Birkerod (Denmark) pilot sites. The degradation capacity increased after adding a concentrated culture of Dehalococcoides (DHC) bacteria at the Welgelegen pilot site, while Birkerod site succeeded by adding lactate and acetate as electron donor (+ 30%) and DHC bacteria culture (+ 78%)^34,35^. These successful experiences corroborated laboratory studies that showed how the biodegradation rate of COCs increases compared to natural attenuation under physical–chemical conditions typical from LEGE environments^36^. Despite the abovementioned successful cases, the influence of variable temperature in the removal capacity has not been studied in detail yet, especially at aquifer scale. In addition, despite COCs have been reported to be degraded, the influence of LEGE on OCECs from urban groundwater remains to be investigated.

So far, the influence of water temperature on the degradation rates of OCECs has only been seriously investigated in river bank filtration cases. In this context, Burke et al.^9,14,37^ observed under controlled laboratory conditions that the degradation rates of different OCECs, such as carbamazepine or phenazone, increase with water temperature. However, these experiments were only performed under two water temperatures. At the field scale, Munz et al.^15^ observed that the variation in the concentration of different OCECs, such as diclofenac, was mostly explained by the seasonal variations of temperature of the infiltrated water. The temperature dependence of OCECs has also been investigated numerically^38,39^. Greskowiak et al.^38^ studied the behaviour of phenazone in a water bank filtration scheme and demonstrated that the evolution of phenazone could only be explained by considering a temperature factor in the degradation. Barkow et al.^39^ numerically simulated the behaviour of different OCECs, such as phenazone and carbamazepine, and concluded that the water temperature is an important factor. Despite these previous relevant investigations, the water temperature range in water bank filtration is lower than that expected around geothermal exploitations (> 45ºC in the case of high temperature aquifer thermal storage systems^40^). In addition, the behaviour of OCECs in a river bank filtration is expected to be different from under the influence of geothermal energy systems. In LEGE, groundwater temperature varies as a result of the energetic requirements, the employed LEGE scheme and its operational characteristics (e.g. the number, location and capacity of production and injection wells, among others).

The behaviour of selected OCECs under the influence of geothermal facilities has only been evaluated by García-Gil et al.^41^ in a qualitative way. The authors concluded, based on field observations, that conditions induced by LEGE contribute to the prevalence of OCECs. However, considering the observed and modelled behaviour of OCECs in river bank filtration contexts and the results of this investigation, the link between LEGE and OCECs observed by García-Gil et al.^41^ could be the result of other factors, such as the population density. In those places where there is more population density, it is probable that there are more LEGE facilities but also that more pollutants reach the aquifer.

This work investigates the influence of LEGE on the behaviour of OCECs in groundwater and the potential of LEGE for bioremediation purposes using reactive transport numerical modelling. To this end, we examine the behaviour of OCECs by considering different LEGE exploitation designs using a reality-based numerical model (i.e., based on real data). The numerical model is based on a study site located in Barcelona (Spain) where a new library building (NLB), with a geothermal facility to satisfy its energetic demands, is planned. The numerical model investigates the impact of LEGE on two OCECs of pharmaceutical origin that are commonly reported in Barcelona´s aquifers, carbamazepine (CBZ) and diclofenac (DCF)^3,42,43^. The considered LEGE scheme is an open-loop type (i.e., a groundwater heat pump—GWHP type) (see the “Methods” Section for more details). We further evaluate the behaviour of OCECs in a LEGE context by using synthetic numerical models (based on artificially generated data). Information relative to the synthetic approach (characteristics of the models, problem statement and results) is summarized in Appendix A of the supplementary material.

## Results

### Concentration and groundwater temperature across the aquifer

**Figure 1:**
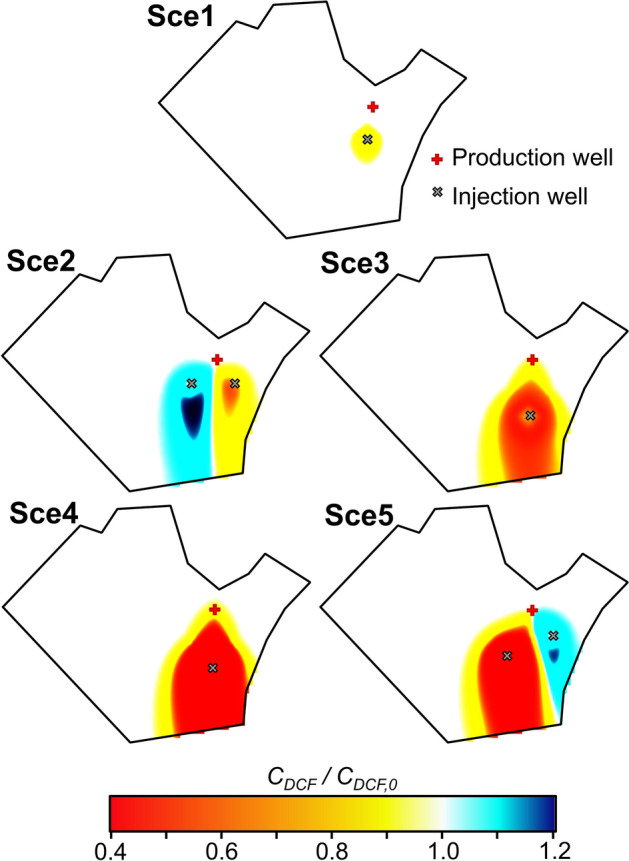
Normalized concentrations of diclofenac predicted for the five considered scenarios (Sce1 to Sce5—see Section “Simulated exploitation scenarios”) after 10 years of operation. The concentration of diclofenac (C_DCF_) is normalized using the concentration of diclofenac under unperturbed conditions (C_DCF,0_).

**Figure 2:**
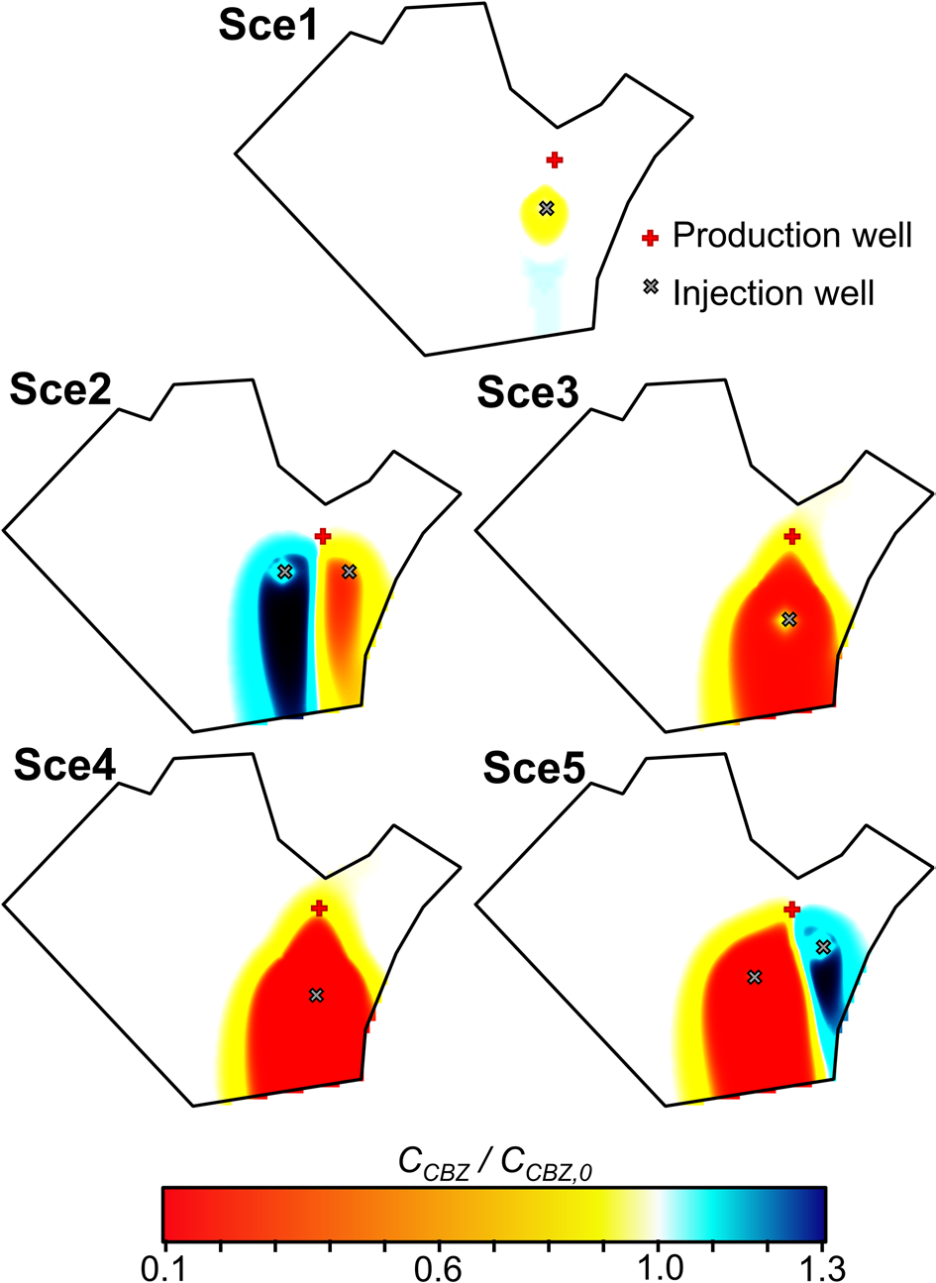
Normalized concentrations of carbamazepine predicted for the five considered scenarios (Sce1 to Sce5—see section “Simulated exploitation scenarios”) after 10 years of operation. The concentration of carbamazepine (C_CBZ_) is normalized using the concentration of carbamazepine under unperturbed conditions (C_CBZ,0_).

**Figure 3:**
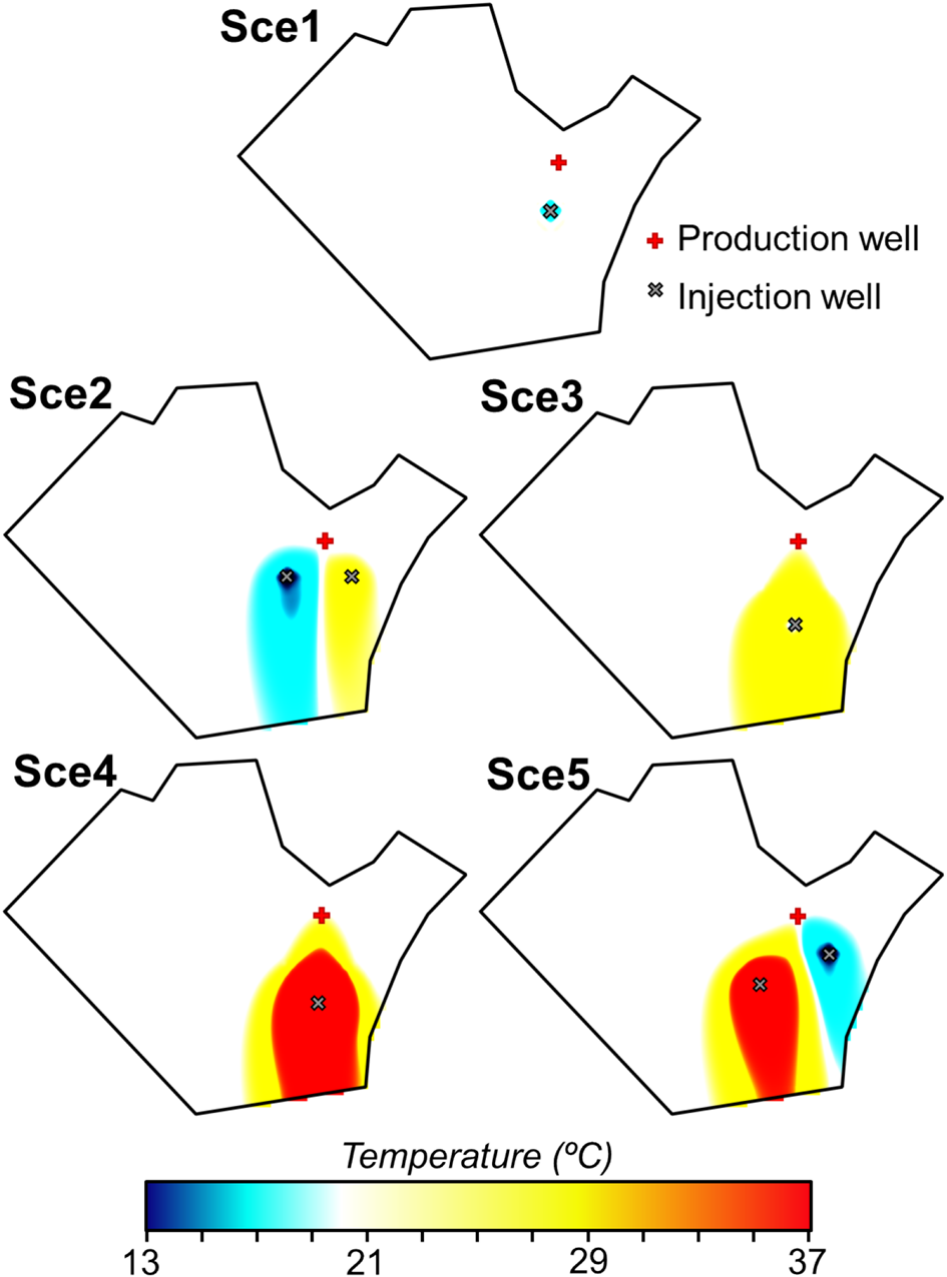
Groundwater temperature predicted for the five considered scenarios (Sce1 to Sce5—see section “Simulated exploitation scenarios”) after 10 years of operation.

Figures 1, 2 and 3 show the normalized concentration of diclofenac and carbamazepine, and groundwater temperature at the end of 5 simulated scenarios (Sce*i*, where *i* is the number of the scenario) in the study site located in Barcelona. The 5 simulated scenarios differ in the pumped and injected flow rates, the number and location of the wells, the uses given to the facility and the energy obtained, but all of them satisfy the minimum energetic requirements of the NLB. The concentration of diclofenac (*C*_*DCF*_) and carbamazepine (*C*_*CBZ*_) are normalized, respectively, by the concentration of diclofenac (*C*_*DCF,0*_) and carbamazepine (*C*_*CBZ,0*_) under unperturbed (i.e., initial) conditions. The first simulated scenario (Sce1) barely modifies the concentrations of the assessed OCECs in the aquifer. Only small variations occur around the injection well. The limited changes occur because variations of groundwater temperature induced by GWHP are low and restricted to the surrounding of the injection well (Fig. 3). In addition, the pumped and injected flow-rates are relatively low, and thus, the volume of mobilised water is low in comparison with that of the aquifer. In the scenario Sce2, small variations with respect to the unperturbed conditions are observed downgradient of the injection wells. The concentration of diclofenac and carbamazepine decreases downgradient of the injection well that introduces hot water and increases downgradient of the well injecting cold water (Fig. 3), which agrees with Eqs. (1) and (3). The normalized concentration of diclofenac at the end of the simulated period decreases up to 0.8 around the hot injection well and increases up to 1.15 around the cold injection well. Similarly, the normalized concentration of carbamazepine decreases down to 0.7 and increases up to 1.3 around the hot and cold injection wells, respectively. The volume of mobilised water seems to be an important factor controlling the impact of GWHP, which is supported by the results of Sce3, Sce4, and Sce5 scenarios, where the pumped and injected flow-rates are doubled. When the flow rate is increased, the volume of groundwater affected by the GWHP facility, and thus, the variations of temperature (Fig. 3) increase. Consequently, the influence of GWHP on the selected OCECs also increases.

The simulated GWHP in the scenario Sce3 reduces the concentration of the chosen OCECs downgradient and around it. This greater reduction of the concentration compared to that observed in Sce1 and Sce2 scenarios occurs because during summer the potential of the groundwater is exploited to the maximum obtaining more energy than that required by the NLB. The injected water during hot months is at 37.5 °C, which produces a plume of hot groundwater (Fig. 3) where the degradation rates of diclofenac and carbamazepine increase.

The largest reductions in the concentrations of the OCECs occur in the scenario Sce4 because the proposed GWHP scheme exploits the cooling potential of the aquifer (i) to the maximum, and (ii) during the whole year. Thus, the temperature of the hot water plume is higher than in the other scenarios (Fig. 3). Despite in the scenario Sce5 the cooling potential of the aquifer is also exploited during the whole year and the hot water is injected through a separate well, the decrease in concentrations is lower than that observed in the scenario Sce4. This fact occurs because the energy obtained for cooling purposes during cold months in the scenario Sce5 is lower than that obtained in the scenario Sce4, and then the size of the hot water plume is smaller than in the scenario Sce4 (Fig. 3). Note that, in the Sce2 and Sce5 scenarios, the normalized concentration of diclofenac and carbamazepine is higher than 1 around and downgradient the cold injection well (i.e., is higher than the concentration under unperturbed conditions). This fact occurs because the groundwater temperature in this area decrease in comparison to unperturbed conditions. Then, the removal rate in this area is lower and the final OCECs concentration is higher than under natural conditions.

The adopted values for the hydraulic conductivity (*K*), the effective porosity (*θ*_*eff*_), the longitudinal and transversal dispersivities (*D*_*L*_* and D*_*T*_) and the thermal diffusivity (*D*_*m*_) can play a significant role in the behavior of OCECs under the influence of GWHP. Although the parameters used in our model have been derived from field investigations, the sensitivity of the model to them has been assessed to investigate their effect on the results. Thus, some additional simulations have been developed by varying the values of *K*, *θ*_*eff*_, *D*_*L*_,* D*_*T*_ and *D*_*m*_. Simulations are developed considering scenario Sce4. This scenario is chosen because it is the most favourable to enhance the degradation capacity against the selected OCECs. The results of these simulations and their discussion are shown in the supplementary material (Appendix B).

### Evolution of concentration and groundwater temperature at the downgradient boundary

**Figure 4:**
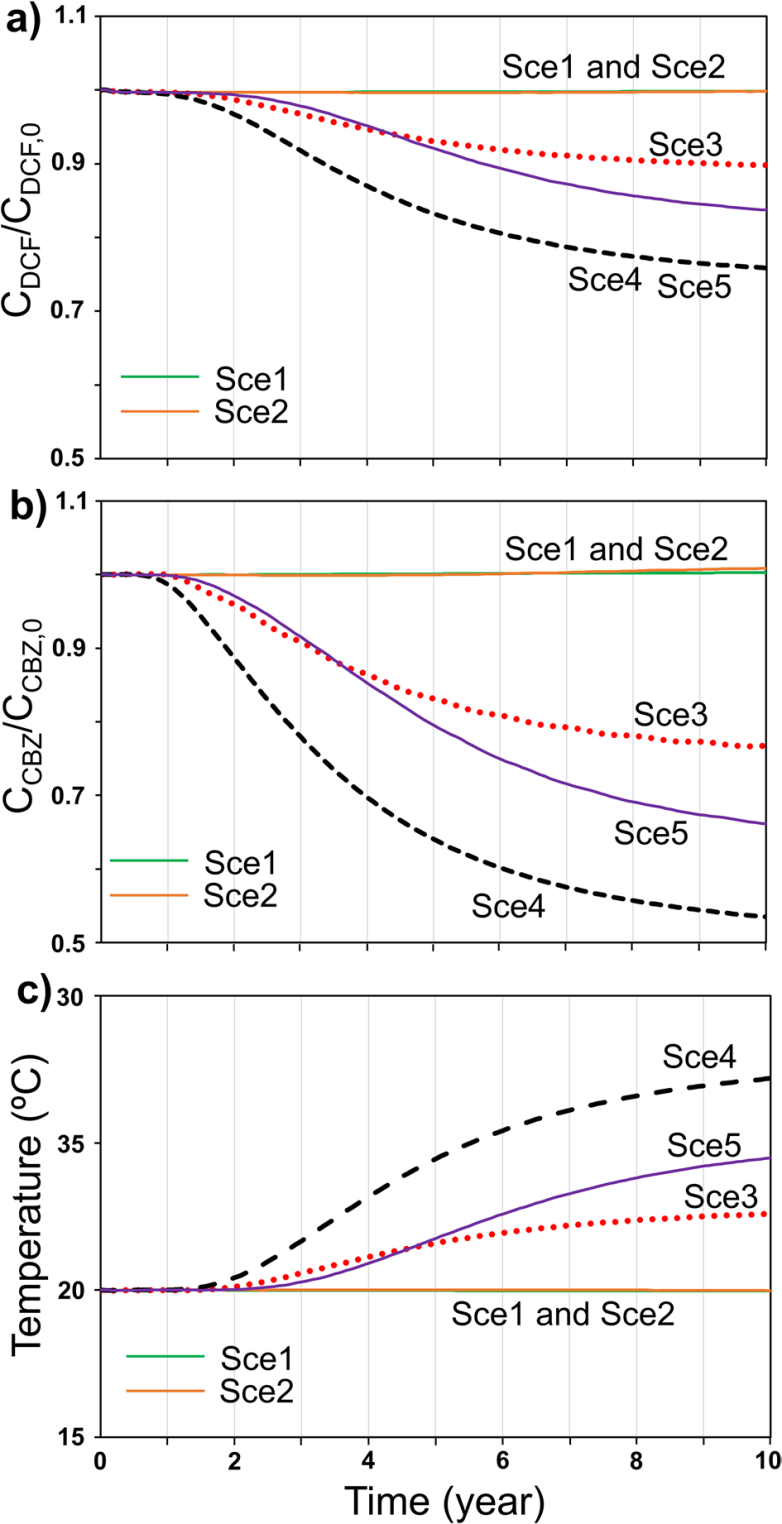
Normalized concentration of (**a**) diclofenac (C_DCF_/C_DCF,0_) and (**b**) carbamazepine (C_CBZ_/C_CBZ,0_), and (**c**) temperature of the groundwater flowing out of the model through the downgradient boundary for the five considered scenarios. Values are computed considering the whole volume of water crossing the boundary.

Similar conclusions can be drawn when observing the concentration of the selected OCECs (carbamazepine and diclofenac) in the water that flows out the aquifer along the downgradient boundary. Figure 4 shows the concentration of diclofenac (Fig. 4a) and carbamazepine (Fig. 4b) through the downgradient boundary normalized by the initial concentration (i.e., under unperturbed conditions). Figure 4c displays the evolution of the groundwater temperature through the downgradient boundary. Results are obtained considering the whole volume of water crossing the boundary. The Sce1 and Sce2 scenarios barely modify the concentration of the studied OCECs. Despite the concentrations of diclofenac and carbamazepine vary in the scenario Sce2, their decrease around and downgradient of the hot well is compensated by their increase (i.e., reduction of the degradation rate) around the cold well. As a result, the concentrations of both OCECs in the scenario Sce2 slightly increase in the downgradient boundary in comparison with the unperturbed conditions. Concentrations significantly decrease for the Sce3, Sce4 and Sce5 scenarios. After 10 years of operation, the normalized concentration of diclofenac decays down to 0.9, 0.76 and 0.84 for the Sce3, Sce4 and Sce5 scenarios, respectively, while that of carbamazepine decreases down to 0.77, 0.54 and 0.67 for the Sce3, Sce4 and Sce5 cases, respectively. It is important to highlight that after 10 years of operation, the concentration of diclofenac and carbamazepine through the downgradient boundary continues to decrease and a steady state is not reached. Thus, the concentration of these OCECs will continue decreasing and the final impact of the GWHP facility will be larger for longer operational times. The evolution of the concentrations at the downgradient boundary agree with the groundwater temperature. Variations of the groundwater temperature are only observed for the Sce3, Sce4, and Sce5 scenarios; while variations occurred under the Sce1 and Sce2 scenarios are negligible. The maximum groundwater temperature, when considering the whole volume of water crossing the downgradient boundary, reaches 27, 25, and 23 °C for the Sce4, Sce5 and Sce3 scenarios, respectively, when considering the whole volume of water crossing the downgradient boundary. As in the synthetic case (see Appendix A of the supplementary material), the observed changes in the concentration of the OCECs in the downgradient boundary start earlier than the groundwater temperature variations. This fact is also related to the difference between the considered retardation factors for the modelled OCECs and the heat.

The main outcome that can be drawn from the simulation results is that GWHP facilities have the potential to modify the concentration of OCECs, as well as organic compounds across the aquifers, and that their impact depends on the facility design. Theoretically, GWHP scenarios that produce large plumes of high temperature reduce in a higher degree the concentration of OCECs. The impact of GWHP is higher for the carbamazepine than for the diclofenac, which is a consequence of the temperature dependency of both compounds. From 20 to 35 °C, the degradation velocity of diclofenac increases by a factor of 2 while it increases by a factor of 5 for carbamazepine.

Unfortunately, numerical results could not be compared with real measurements because the site is still under construction and the geothermal facility has not been built yet. Once the construction will be finished, it is planned to periodically take groundwater samples upgradient and downgradient the study site to analyse the behaviour of OCECs under the influence of the geothermal facility.

## Discussion

This research represents a step forward in the field of urban water resources and groundwater remediation, as it shows that LEGE facilities can significantly modify the concentration and distribution of OCECs. The comparison between different GWHP scenarios indicates that the facility design (given uses, energy production or position of wells) plays a critical role in the behaviour of OCECs, increasing their attenuation as the thermal plume becomes larger. This fact suggests that properly designed GWHP facilities have the potential to improve the quality of groundwater by degrading OCECs, and thus increasing the quality and amount of available freshwater resources.

The variations observed in the concentration of OCECs as a result of the simulated GWHP facilities can be considered as relatively low, in the order of ng L^−1^. However, it should be borne in mind that hundreds, or even thousands, of different OCECs can be found in urban aquifers^44^. Therefore, the global impact of GWHP facilities towards OCECs attenuation will be much larger, substantially improving the quality of groundwater resources. In addition, the two considered OCECs (carbamazepine and diclofenac) have low retardation factors. Thus, it is expected that GWHP impact will increase in other OCECs with high retardation factors, as the residence time within the thermal plume will be longer. However, the increase of groundwater temperature induced by GWHP could have counter-productive effects under some circumstances, especially when transformation products are more persistent, mobile, and harmful (enhanced toxicity) than their parent compound^45^. Therefore, it will be needed to analyse the nature of the potential transformation products at any specific case to evaluate the benefits and disadvantages, in terms of groundwater quality, of installing a LEGE facility with remediation purposes.

The GWHP scenarios presented in this investigation are designed to take account of their utilization in a maritime Mediterranean climate (Köppen-Geiger classification: Csa^46^), like in Barcelona (Spain), where cooling requirements are usually higher than heating ones. Thus, the volume of hot water introduced in the aquifer is expected to be higher than that of cold water. Instead, if GWHP facilities are used mainly for heating purposes, the volume of injected cold water will increase, having negative consequences regarding the groundwater quality because the degradation rate of OCECs will decrease.

A factor that may improve bioremediation in urban aquifers is subsurface urban heat islands (SUHI). The temperature increase of a few degrees associated with SUHI will increase the degradation rates of OCECs improving the groundwater quality. The contribution of SUHI to improve bioremediation of urban aquifers deserves to be deeply investigated since many anthropogenic OCECs reach aquifers in urban areas, where SUHI occur.

Finally, it is needed to highlight that although the impact on groundwater temperature of GWHP seems to be beneficial in terms of groundwater quality, there are some issues that deserve further investigation. For example, it is necessary to reach an agreement between the reduction of OCECs and the potential negative impacts related to the creation of a large thermal plume that could affect the efficiency of LEGE facilities located downgradient. LEGE design at the city scale should take into consideration upgradient LEGE facilities. For example, a LEGE downgradient of the LEGEs considered in scenarios Sce 2 and 5 could improve its performance by drilling two pumping wells, taking water from the cold plume for cooling and water from the hot plume for heating. Consequently, the design of LEGE facilities should include the influence of aquifer properties on temperature variations and the interactions between adjacent LEGEs. In addition, it is needed to consider biodegradation potential in the presence of a wide range of OCECs and under variable redox conditions. In this regard, changes of aquifer temperature can have a critical effect on various (a)biotic processes like microbial activity, redox (electron transfer reactions), pH, as well as contaminant transport and fate (e.g. (co)precipitation-dissolution, adsorption–desorption, (bio)transformation). For instance, an increase in aquifer temperature could foster consumption of dissolved oxygen and organic carbon concentrations by microbial activity, resulting in suboxic/anoxic conditions in the aquifer. Additionally, these low redox conditions could dissolve manganese and iron oxides, contributing to the mobility of arsenic (As) turbidity or clogging^47^. In any case, attaining sulphate-reducing conditions could release sulphide ion which can be toxic and corrosive^48^.

## Methods

### Geothermal scheme

There are two types of ground source heat pumps (GSHPs), closed-loop systems, where the heat is exchanged with the ground through the circulation of a carrier fluid in borehole heat exchanger (BHE) buried into the ground^49,50^, and open-loop systems, also named groundwater heat pumps (GWHPs)^51,52^. In GWHP schemes, groundwater is pumped from aquifers and carried to surface heat exchangers, where heat is exchanged with the working fluid of a heat pump. Subsequently, water is commonly returned to the aquifer through a discharge well^53^. In this study, we consider GWHP schemes since these systems are less costly and more efficient^50^ than GSHP schemes when clean water is available^54^. Clean groundwater refers to main quality standards, such as pH, hardness, iron content, dissolved oxygen and turbidity, because if these parameters are not acceptable, as previously noted, corrosion, incrustation, erosion or clogging may occur^55^. Thus, despite deterioration of urban groundwater quality due to the presence of OCECs, for example for potable water, this is not significant for water quality standards for GWHP.

### General description of the modelled site

The influence of GWHP on selected OCECs is investigated using a model based on a real site. The site, which is located in Barcelona (Spain), is an industrial complex where an important textile factory, named “Can Batlló”, which was operative from 1880 to 1964. Currently, it is planned to construct green areas and public facilities in the area occupied by the main factory and the adjacent industrial units. One of the planned actions is to transform the old main existing building of the factory to build a new modern library building (NLB). In accordance with the policies and commitment of the Barcelona City Council to climate change mitigation, it is planned to use LEGE to provide heating and cooling to the future NLB. In this context, some previous investigations, which only have addressed the problem from an energy point of view, have been developed to establish the viability of different LEGE scenarios^56^. As a result of these investigations, it has been decided to use a closed-loop type geothermal facility to cover only the heating and cooling requirements of the NLB. Here, we want to go further and give a vision about the potential of a hypothetical LEGE facility of the open-loop type (i.e., GWHP) to cover the energetic demand and to improve the quality of groundwater by enhancing the removal of OCECs.

### Geographical, geological and hydrogeological context

**Figure 5:**
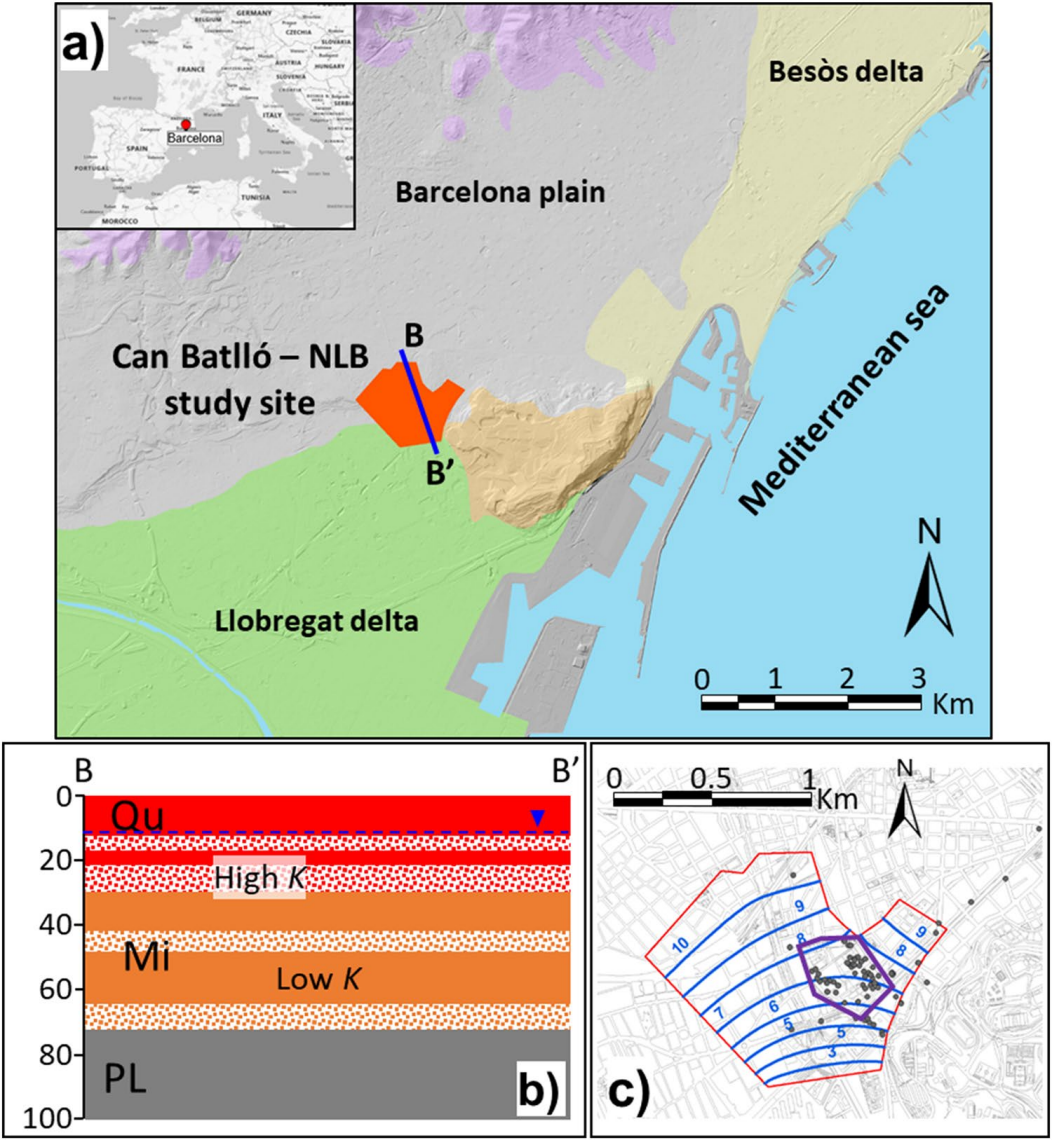
(**a**) Location map with general geological materials at the study site (named as “Can Batlló”): Cenozoic (grey), Mesozoic deltaic materials: Llobregat (green) and Besòs (yellow) deltas, and Paleozoic materials (purple). (**b**) Geological cross-section: Quaternary -Qu (red), Miocene—Mi (orange) and Pliocene—PL (grey) materials. Two textures are used for differentiating the lithology. Totally colored layers represent materials with low hydraulic conductivity (i.e., clay or silt) while dotted layers represent materials with a relative high hydraulic conductivity (i.e., sand and gravel). (**c**) Piezometry at the study site (blue lines). Grey dots represent the locations where the piezometric head has been measured, the red line indicates the modeled area and the purple line highlights the place where the new library building (NLB) is planned. The piezometric head is representative of Quaternary and Miocene materials since they can be considered as a single multilayer aquifer (background map source OpenStreetMap).

The study site (Can Batlló) is located in Barcelona (North-East of Spain). The site is placed in the Barcelona plain between the deltas of the Besòs (North-East) and Llobregat (South-West) rivers (Fig. 5a). The Barcelona plain is also surrounded by the Collserola mountain range (West and North-West) and the Mediterranean Sea (East and South-East) (Fig. 5a).

Geologically, Can Batlló is located at the intersection of three sedimentary units (Fig. 5b). These units are: (1) the Barcelona plain; (2) the Montjuïc deposits and (3) the Llobregat delta river unit. Specifically, Can Batlló is found between 3 paleochannels of Quaternary age (Fig. 5c). From borehole information and collected data, Miocene, Pliocene and Quaternary materials can be distinguished below the study site (Fig. 5b). Quaternary materials are between 10 and 30-m thick and largely correspond to the filling deposits of the Barcelona plain and Montjuïc streams. These Quaternary materials consist in clay and silt deposits intercalated with layers of sand and gravel. The Miocene materials are deposits of the Serravallian (Middle Miocene) and they reach up to 40 m of thickness. They consist of clay and marl deposits intercalated with layers of sand and gravel. Finally, two types of Pliocene materials can be distinguished in Can Batlló: (1) yellowish silt and (2) bluish marl. Both are made of compacted, slightly permeable and slightly cemented materials, which form part of the Llobregat river delta basement. Hydrogeologically, according to the piezometric map (Fig. 5c), the flow direction depends on the three Quaternary paleochannels. The water table is located in the Quaternary materials between 6 and 8 m.a.s.l. The average hydraulic gradient ranges from 0.005 to 0.006 and the groundwater flows towards the South-East. Hydraulic tests carried out in the surroundings reveal an average effective hydraulic conductivity ($K$) of 3.3 m d^−1^ for the Quaternary and Miocene materials that can be considered as a single multilayer aquifer.

### Presence of OCECs and hydrochemical conditions of the study site

The presence of OCECs has been reported in the study site through a sampling campaign conducted in 2021 at several observation points located in the area. A sample taken in the proximity of the future NLB revealed the presence of 81 compounds (Appendix C in the supplementary material). The pharmaceuticals diclofenac and carbamazepine are selected for analysing the impact of GWHP because data on their behaviour at different temperatures is available in the literature^14,39^. In addition, both contaminants differ in the redox conditions that enhance their degradation. Degradation of carbamazepine is enhanced under low concentrations of oxygen and nitrate^15,38,57^, while that of diclofenac increases under oxic conditions^24^. The measured concentrations of diclofenac and carbamazepine were 21.3 and 13.9 ng L^−1^, respectively. Groundwater is sub-oxic with low concentrations of dissolved oxygen (≈0.5 mg L^−1^; 1.6·10^–5^ mol L^−1^), and high concentrations of nitrate (≈60 mg L^−1^; 9.7·10^–4^ mol L^−1^) and sulphate (≈200 mg L^−1^; 2.1·10^−3^ mol L^−1^).

### Heating and cooling requirements

**Figure 6:**
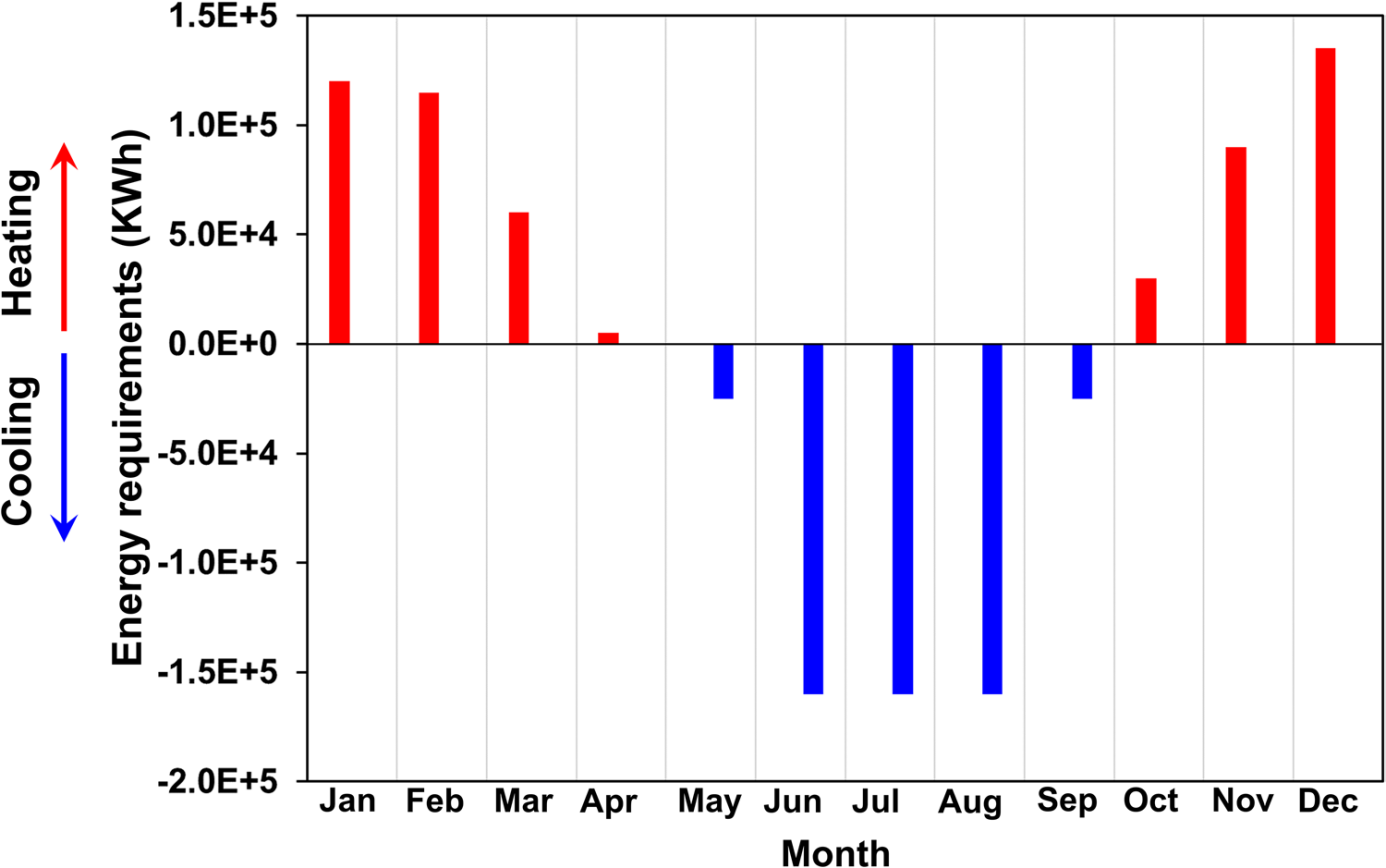
Net monthly energy required for temperature regulation (i.e., heating and cooling) of the future NLB. Positive values (red bars) refer to energy needed for heating while negative ones (blue bars) refer to energy required for cooling.

The minimum energetic requirements for the NLB has been calculated considering the dimensions and uses of the conditioning system (Fig. 6). The maximum net monthly energy required for heating is 140 MWh (in winter), and for cooling is 165 MWh (in summer).

### Numerical approach

PHT3D^58^ code was used to build the numerical model. This code solves advective‐dispersive‐reactive transport processes by coupling MT3DMS with PHREEQC^59^. The numerical model simulates the Quaternary and Miocene materials because (i) the Pliocene formation has a low hydraulic conductivity, and (ii) the hypothetical GWHP system would be located in these formations. Quaternary and Miocene sediments are considered and modelled as a single aquifer with $K$ of 3.3 m d^−1^ because they comprise similar deposits (i.e., clay, marl or silt deposits intercalated with layers of sand and gravel). The hydraulic and transport parameters (compounds and heat) have been derived from field tests (5 pumping tests and 2 thermal response tests), and considering the lithology of the materials (Table 1).

**Table 1:** Aquifer properties at the study site (Can Batlló).

Parameter	Value
Hydraulic conductivity (*K*)	3.3 m d^−1^
Anisotropy factor	1
Specific storage (S_S_)	0.0017 m^−1^
Effective porosity (*θ*_*eff*_)	0.04
Longitudinal dispersion (*D*_*L*_)	25 m
Transverse dispersion (*D*_*T*_)	10 m
Thermal diffusivity (*D*_*m*_)	1.86·10^−6^ m^2^d^−1^
Heat capacity (${S}_{VCaq}$)	2.09 MJ m^−3^ K^−1^
Thermal conductivity (λ)	1.92 W mK^−1^
Boundary conductance	0.4 m d^−1^

Values are derived from pumping tests and thermal response tests performed at the site.

The value of *θ*_*eff*_ is calculated by considering the measured porosity for the more permeable layers (0.1) and their total thickness (20 m) in comparison with the whole thickness of the simulated materials (60 m).

The thermal diffusivity coefficient is selected in agreement with the reference values specified in the guidelines for thermal use of the underground of the German Engineer Association^60^.

**Figure 7:**
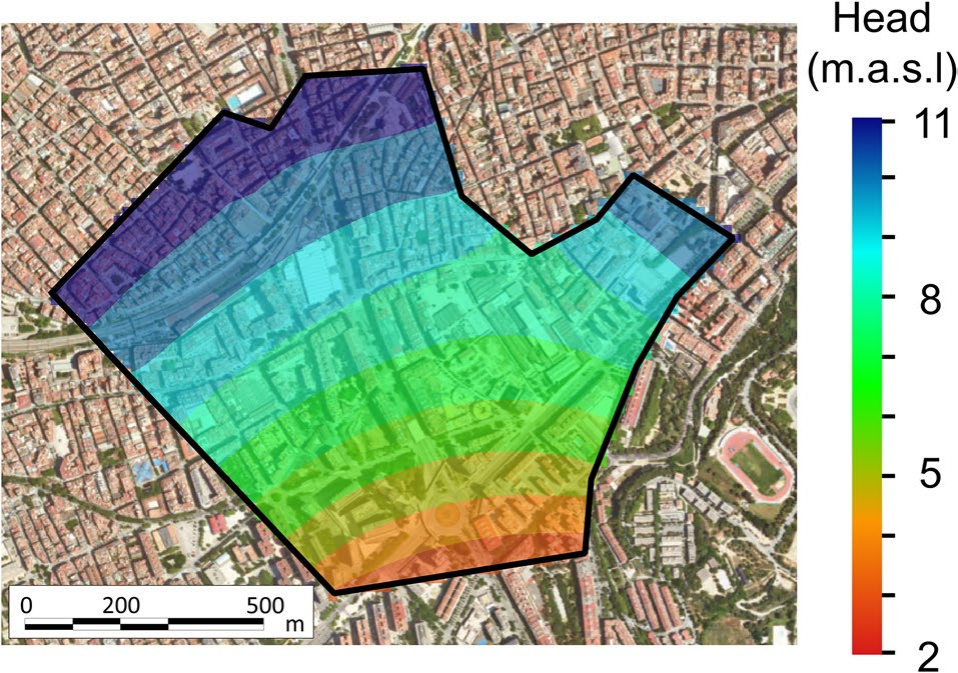
Calculated initial conditions based on field measurements of the hydraulic head.

The model consists of one layer divided in 2628 regular cells with an area of 400 m^2^ (20 × 20 m) each. The simulation period covers 10 years with a 10-day time step to solve the flow problem and a 1-day step for the transport problem. Boundary conditions (BCs) have been chosen in accordance with the hydrogeological behaviour of the site and the observed concentrations of OCECs. Flow BCs consist in a mixed BC at the upgradient and downgradient boundaries. The general head BC is implemented to minimize the influence of the BC on the results. The applied BC conductance (0.4 m d^−1^) is calculated by assuming that during the simulation the piezometric head does not vary 500 m beyond the current boundaries. The prescribed head on the upgradient and downgradient boundaries is chosen to reproduce the observed piezometry under unperturbed conditions (Figs. 5c and 7). The production and injection wells are modelled by using Neumann BCs. Concerning the transport BCs, the concentration of OCECs is prescribed on the upgradient boundaries according to the measured concentrations at the field. Additionally, a constant input of mass of 6.1·10^−12^ and 1.1·10^−12^ mol d^−1^ per square meter is imposed in the whole domain for diclofenac and carbamazepine, respectively, to mimic the observed concentrations in the aquifer.

Diclofenac and carbamazepine are modelled using the Monod kinetics and considering the redox conditions that enhance their degradation. The degradation of carbamazepine is modelled as the 1st order Monod kinetics including an inhibition term to account for the dissolved oxygen concentration (O_2_). The carbamazepine degradation rate (${r}_{CBZ}$) is modelled as

1 $${r}_{CBZ}=-{\lambda }_{CBZ}^{MX}{C}_{CBZ}\frac{{K}_{{inhO}_{2}}}{{K}_{{inhO}_{2}}+{C}_{{O}_{2}}}{f}_{T},$$

where ${\lambda }_{CBZ}^{MX}$ is the maximum degradation rate constant of carbamazepine, ${C}_{CBZ}$ is the concentration of carbamazepine, ${K}_{{inhO}_{2}}$ is the inhibition coefficient for O_2_, ${C}_{{O}_{2}}$ is the concentration of O_2_, and ${f}_{T}$ is a function that depends on the temperature. ${f}_{T}$ is defined with the Arrhenius equation adding a normalization factor ($\beta$). $\beta$ allows normalizing the result to 1 when the temperature of groundwater is between 35 and 40 °C, which is when the highest microorganism activity occurs^38,61–63^, as

2 $${f}_{T}=\beta A{e}^{-\frac{{E}_{A}}{RT}},$$

where *A* is a pre-exponential factor, *E*_*A*_ is the activation energy, *R* is the gas constant and *T* the temperature in Kelvin. Similarly, the degradation of diclofenac was approximated as a 1st order degradation. In this case, a Monod term to incorporate the influence of O_2_^39^ is included. The diclofenac degradation rate (${r}_{DCF}$) is modelled as:

3 $${r}_{DCF}=-{\lambda }_{DCF}^{MX}{C}_{DCF}\frac{{C}_{{O}_{2}}}{{K}_{{O}_{2}}+{C}_{{O}_{2}}}{f}_{T},$$

where ${K}_{{O}_{2}}$ is the Monod half-saturation constant of diclofenac, ${\lambda }_{DCF}^{MX}$ is the maximum degradation rate constant of diclofenac, and ${C}_{DCF}$ is the concentration of diclofenac. Parameters used in Eqs. (1) to (3) are obtained from bibliographical data (Table 2). Parameters for computing ${f}_{T}$ (*A* and *E*_*A*_) are derived from laboratory data provided for carbamazepine by Burke et al.^14^. ${\lambda }_{CBZ}^{MX}$ and ${\lambda }_{DCF}^{MX}$ are obtained from Barkow et al.^39^. ${K}_{{inhO}_{2}}$ is obtained by fitting Eq. (1) to ensure that ${r}_{CBZ}$ is equal to ${\lambda }_{CBZ}^{MX}$ in the absence of oxygen and, according to Regnery et al.^64^, very low (0.001 d^−1^) under oxic conditions (O_2_ ≥ 1 mg L^−1^). Low removal rate under aerobic conditions has been corroborated by several authors^15,65–67^. ${K}_{{O}_{2}}$ is calculated by fitting Eq. (3) to ensure that ${r}_{DCF}$ matches ${\lambda }_{DCF}^{MX}$ under aerobic conditions and is equal to 0.03 d^-1^ under sub-oxic/anoxic conditions. This value has been calculated by averaging data from Banzhaf et al.^68^ and Heberer et al.^69,70^ summarized in Henzler et al.^65^. Despite the retardation factors of carbamazepine and diclofenac are low^57,65^, we consider them to increase the accuracy of the results with a value of 1.9 and 1.41 for the carbamazepine and diclofenac, respectively^71^.

**Table 2:** Parameters for carbamazepine and diclofenac.

Parameter	Carbamazepine	Diclofenac
Pre-exponential factor (*A*)	1.78·10^9^ s^−1^	3.37 s^−1^
Activation energy (*E*_*A*_)	8.30·10^4^ J mol^−1^	2.49·10^4^ J mol^−1^
Normalizing factor ($\beta$)	66,373	5011
Maximum degradation rate constant (${\lambda }_{CBZ}^{MX}$)	1.3·10^−6^ s^−1^	2·10^−6^ s^−1^
Inhibition coefficient (${K}_{{inhO}_{2}}$)	1.5·10^−6^ mol L^−1^	3·10^−5^ mol L^−1^

### Simulated exploitation scenarios

**Figure 8:**
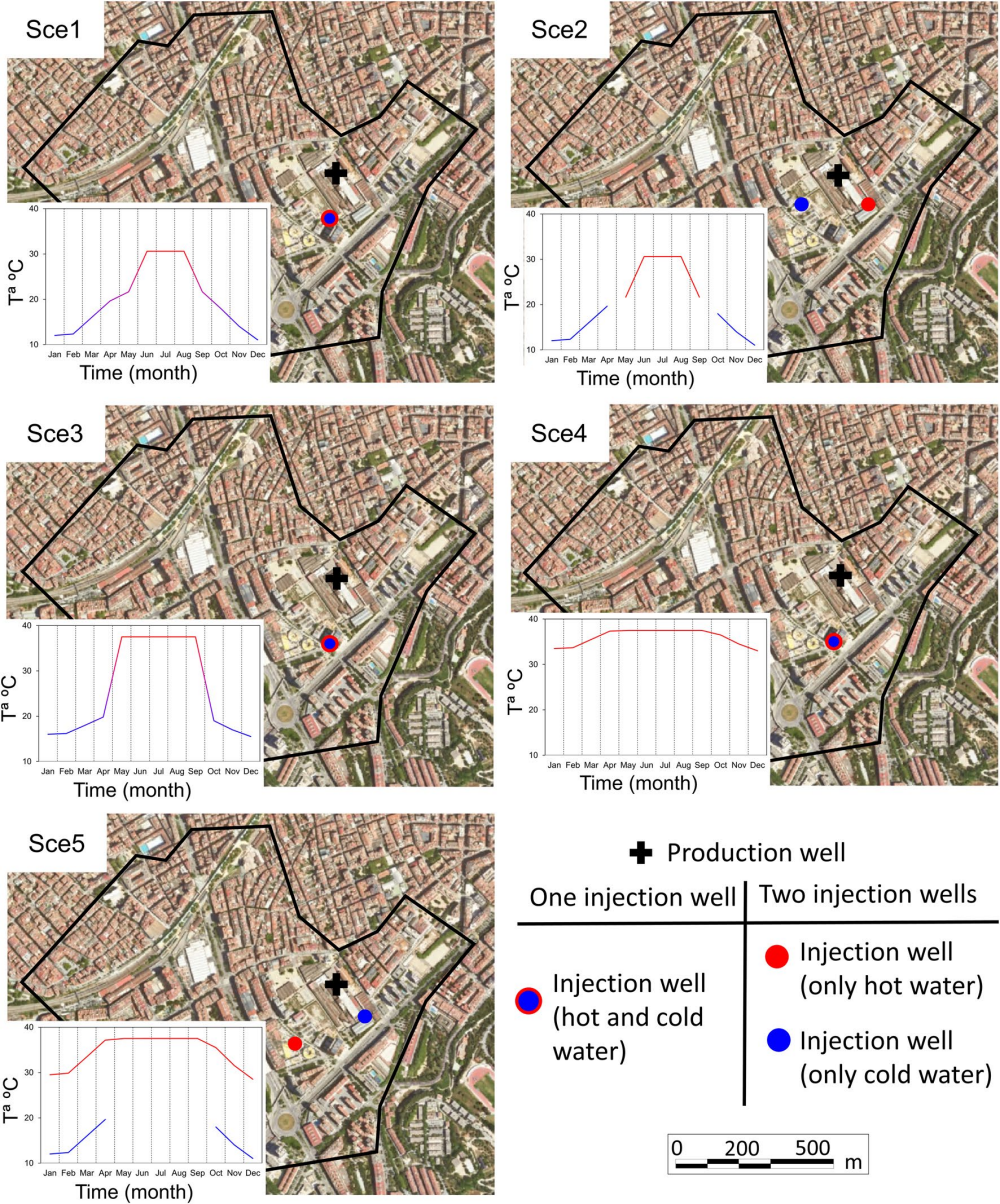
Location of the pumping and injection wells and temperature of the injected water for all scenarios.

Five different hypothetical scenarios that satisfy the energetic requirements of the NLB are simulated to compute their impact on OCECs distribution. They differ in the pumped and injected flow rates, the number and location of the wells and the uses given to the facility. The distance between wells (production and injection) is calculated to avoid thermal breakthrough (*t*_*BR*_) during the simulated period according to^72^

4 $${t}_{BR}=\frac{{S}_{VCaq}L}{{S}_{VCwat}Ki}\left[1+\frac{4\alpha }{\sqrt{-1-4\alpha }} {tan}^{-1}\left(\frac{1}{\sqrt{-1-4\alpha }}\right)\right],$$

where ${S}_{VCaq}$ is the volumetric heat capacity of the aquifer (2800 J kg^−1^ K^−1^; Ref.^73^, ${S}_{VCwat}$ is the volumetric heat capacity of the water at 20 °C (4180 J kg^−1^ K^−1^), $L$ is the distance between wells and $\alpha$ is defined as

5 $$\alpha =\frac{Q}{2\pi KbiL},$$

where *b* is the saturated thickness and *Q* is the pumping/injection rate. The temperature of injected water in the models is computed according to the thermal potential of the system (*P*_*GW*_) as follows:

6 $${P}_{GW}={S}_{VCwat}Q\Delta T,$$

where $\Delta T$ is the temperature difference between the production and injection wells. The groundwater temperature under unperturbed conditions is assumed constant and equal to 20 °C^74^. Scenarios 3, 4 and 5 consider the possibility of obtaining more energy than needed to maximize the usefulness of the GWHP facility. The considered scenarios are as follows:

- Scenario 1 (Sce1): This scheme provides only the required energy for the climatization of the NLB. The GWHP system is made up by one production and one injection well (Fig. 8). The pumped and injected flow rates are constant and equal to 432 m^3^ d^−1^.
- Scenario 2 (Sce2): This scheme provides only the required energy for the climatization of the NLB. The GWHP system is made up by one production and two injection wells (Fig. 8). The pumped and total injected flow rates are constant and equal to 432 m^3^ d^−1^. The two injection wells do not inject water at the same time. One is activated when the facility is used for heating (i.e., cold water is injected) while the other is activated when the facility is used for cooling (i.e., hot water is injected).
- Scenario 3 (Sce3): This scheme provides the required heating energy for the climatization of the NLB during cold periods, while during hot periods, more cooling energy than that needed by the NLB is extracted with the objective of providing cooling energy to neighbourhood buildings, factories or other nearby infrastructures. The obtained cooling energy during hot periods is the one that yields an injection temperature of 37.5 °C. This scenario consists in two wells (one for production and one for injection) and the pumped and injected flow rates are of 864 m^3^ d^−1^ (Fig. 8).
- Scenario 4 (Sce4): This scheme considers the possibility of providing, in addition to the heating and cooling energy needed by the NLB, continuous cooling energy to neighbourhood factories and infrastructures that need it, such as data centres containing high-performance computing systems or information technology equipment^75^. This scenario consists in two wells (one for pumping and one for injection) (Fig. 8) that pump and inject 864 m^3^d^−1^ and two heat exchangers. During cold months, the needed heating energy is extracted from 30% of the pumped water using one of the heat exchangers, while the rest of the pumped water (70%) is used to produce cooling energy in a second heat exchanger. The extracted energy for cooling is equal to that obtained by the system during hot periods (i.e., 527 MWh). Outflow water from both heat exchangers is mixed and injected into the aquifer through the same well. The maximum temperature of injected water reached during cold months is 36.5 °C. During hot periods, energy for cooling is extracted from the whole pumped water using only one heat exchanger, and the extracted energy is that for which the temperature of the injected water is 37.5 °C.
- Scenario 5 (Sce5): This scheme also considers the possibility of providing, in addition to the heating and cooling energy needed by the NLB, continuous cooling energy to neighbourhood factories and infrastructures^75^. Differently from scenario Sce4, Sce5 consists in three wells (one for pumping and two for injection) and two heat exchangers (Fig. 8). The production well pumps 864 m^3^d^−1^. During hot periods, all pumped water is used for cooling, the extracted energy is that for which the temperature of the injected groundwater is 37.5 °C (i.e., 527 MWh, higher than the demand of the NLB) and the hot water is returned to the aquifer through the hot injection well. During cold months, required heating energy by the NLB is obtained from the half of the pumped water (i.e., 432 m^3^d^−1^) and the resulting cold water from the heating process is returned to the aquifer through the cold injection well. The other half of pumped groundwater (432 m^3^d^−1^) is used to obtain cooling energy. The extracted energy for cooling during these cold periods is that for which the variation induced in the water temperature is 17.5 °C (i.e., 264 MWh) and the resulting hot water is injected through the hot well (Fig. 8).

The excess of energy obtained at Sce2, Sce4 and Sce5 scenarios could be shared (i.e., commercialized) with nearby buildings, factories or other infrastructures like data centres containing information technology equipment, which will contribute to maximize the efficiency of the installation. Considering that Barcelona is located in a maritime Mediterranean climate region (Köppen-Geiger classification: Csa^46^), where atmospheric temperature can reach up to 39 °C in summer^76^, and the demand for cooling is high, this sharing adds value to the installation. Table 3 summarizes the energy obtained from the GWHP facility for each scenario and Fig. 8 displays the location of the wells and the temperature of the injected water at the considered scenarios.

**Table 3:** Energy obtained from the GWHP facility for the considered exploitation scenarios.

Month	Sce1–2 (MWh)		Sce3 (MWh)		Sce4 (MWh)		Sce5 (MWh)	
	Heating	Cooling	Heating	Cooling	Heating	Cooling	Heating	Cooling
Jan	120		120		120	− 527	120	− 143
Feb	115		115		115	− 527	115	− 148
Mar	60		60		60	− 527	60	− 203
Apr	5		5		5	− 527	5	− 258
May		− 25		− 527		− 527		− 527
Jun		− 160		− 527		− 527		− 527
Jul		− 160		− 527		− 527		− 527
Aug		− 160		− 527		− 527		− 527
Sep		− 25		− 527		− 527		
Oct	30		30		30	− 527	30	− 233
Nov	90		90		90	− 527	90	− 173
Dec	135		135		135	− 527	135	− 128

## Supplementary Information

Supplementary Information.

## Data Availability

Data generated or analysed during this study are included in the article/supplementary material, further inquiries can be addressed to the corresponding author.

## References

[1] United Nations, Department of Economic and Social Affairs, & Population Division. *World urbanization prospects: the 2018 revision*. (2019).

[2] Lapworth DJ (2019). Developing a groundwater watch list for substances of emerging concern: A European perspective. Environ. Res. Lett..

[3] López-Serna R (2013). Occurrence of 95 pharmaceuticals and transformation products in urban groundwaters underlying the metropolis of Barcelona, Spain. Environ. Pollut. Barking Essex.

[4] Arnold KE, Brown AR, Ankley GT, Sumpter JP (2014). Medicating the environment: Assessing risks of pharmaceuticals to wildlife and ecosystems. Philos. Trans. R. Soc. B.

[5] Tran NH, Urase T, Ngo HH, Hu J, Ong SL (2013). Insight into metabolic and cometabolic activities of autotrophic and heterotrophic microorganisms in the biodegradation of emerging trace organic contaminants. Bioresour. Technol..

[6] Jurado A, Vázquez-Suñé E, Pujades E (2021). Urban groundwater contamination by non-steroidal anti-inflammatory drugs. Water.

[7] Greskowiak J, Hamann E, Burke V, Massmann G (2017). The uncertainty of biodegradation rate constants of emerging organic compounds in soil and groundwater—A compilation of literature values for 82 substances. Water Res..

[8] Barbieri M (2011). Microcosm experiments to control anaerobic redox conditions when studying the fate of organic micropollutants in aquifer material. J. Contam. Hydrol..

[9] Burke V (2014). Temperature dependent redox zonation and attenuation of wastewater-derived organic micropollutants in the hyporheic zone. Sci. Total Environ..

[10] Massmann G, Dünnbier U, Heberer T, Taute T (2008). Behaviour and redox sensitivity of pharmaceutical residues during bank filtration—Investigation of residues of phenazone-type analgesics. Chemosphere.

[11] Ma Y, Modrzynski JJ, Yang Y, Aamand J, Zheng Y (2021). Redox-dependent biotransformation of sulfonamide antibiotics exceeds sorption and mineralization: Evidence from incubation of sediments from a reclaimed water-affected river. Water Res..

[12] Rodríguez-Escales P, Sanchez-Vila X (2016). Fate of sulfamethoxazole in groundwater: Conceptualizing and modeling metabolite formation under different redox conditions. Water Res..

[13] Liu Y-S, Ying G-G, Shareef A, Kookana RS (2013). Biodegradation of three selected benzotriazoles in aquifer materials under aerobic and anaerobic conditions. J. Contam. Hydrol..

[14] Burke V, Greskowiak J, Grünenbaum N, Massmann G (2017). Redox and temperature dependent attenuation of twenty organic micropollutants—A systematic column study. Water Environ. Res..

[15] Munz M, Oswald SE, Schäfferling R, Lensing H-J (2019). Temperature-dependent redox zonation, nitrate removal and attenuation of organic micropollutants during bank filtration. Water Res..

[16] Kaandorp VP, Doornenbal PJ, Kooi H, Peter Broers H, de Louw PGB (2019). Temperature buffering by groundwater in ecologically valuable lowland streams under current and future climate conditions. J. Hydrol. X.

[17] Bucci A, Barbero D, Lasagna M, Forno MG, De Luca DA (2017). Shallow groundwater temperature in the Turin area (NW Italy): Vertical distribution and anthropogenic effects. Environ. Earth Sci..

[18] Tissen C, Benz SA, Menberg K, Bayer P, Blum P (2019). Groundwater temperature anomalies in central Europe. Environ. Res. Lett..

[19] Hemmerle H (2019). Estimation of groundwater temperatures in Paris, France. Geofluids.

[20] Arola T (2019). Creating Shallow Geothermal Potential Maps for Finland.

[21] Cappellari D, Piccinini L, Pontin A, Fabbri P (2023). Sustainability of an open-loop GWHP system in an Italian Alpine Valley. Sustainability.

[22] Alcaraz M, García-Gil A, Vázquez-Suñé E, Velasco V (2016). Use rights markets for shallow geothermal energy management. Appl. Energy.

[23] Lund JW, Toth AN (2021). Direct utilization of geothermal energy 2020 worldwide review. Geothermics.

[24] García-Gil A (2020). Governance of shallow geothermal energy resources. Energy Policy.

[25] Pekárová P, Tall A, Pekár J, Vitková J, Miklánek P (2022). Groundwater temperature modelling at the water table with a simple heat conduction model. Hydrology.

[26] Drijver, B. & Willemsen, A. Groundwater as a heat source for geothermal heat pumps. In *International summer school on direct application of geothermal energy, International Geothermal Association* pp. 17–20 (2001).

[27] Cui P, Man Y, Fang Z, Yan J (2014). Geothermal heat pumps. Handbook of Clean Energy Systems.

[28] Ahmed AA, Assadi M, Kalantar A, Sliwa T, Sapińska-Śliwa A (2022). A critical review on the use of shallow geothermal energy systems for heating and cooling purposes. Energies.

[29] Zhang Q (2019). Recoverable resource prediction of shallow geothermal energy in small towns using the finite volume method: Taking the Central Urban Area of Danyang City, Jiangsu province, as an example. Math. Probl. Eng..

[30] Saito T (2016). Temperature change affected groundwater quality in a confined marine aquifer during long-term heating and cooling. Water Res..

[31] Allen A, Milenic D, Sikora P (2003). Shallow gravel aquifers and the urban ‘heat island’ effect: A source of low enthalpy geothermal energy. Geothermics.

[32] Slenders HLA, Dols P, Verburg R, de Vries AJ (2010). Sustainable remediation panel: Sustainable synergies for the subsurface: Combining groundwater energy with remediation. Remediat. J..

[33] Sommer WT (2013). Combining Shallow Geothermal Energy and Groundwater Remediation.

[34] Hoekstra N (2020). Increasing market opportunities for renewable energy technologies with innovations in aquifer thermal energy storage. Sci. Total Environ..

[35] Pellegrini M (2019). Low carbon heating and cooling by combining various technologies with aquifer thermal energy storage. Sci. Total Environ..

[36] Ni Z, van Gaans P, Smit M, Rijnaarts H, Grotenhuis T (2016). Combination of aquifer thermal energy storage and enhanced bioremediation: Resilience of reductive dechlorination to redox changes. Appl. Microbiol. Biotechnol..

[37] Burke V (2018). Trace organic removal during river bank filtration for two types of sediment. Water.

[38] Greskowiak J, Prommer H, Massmann G, Nützmann G (2006). Modeling seasonal redox dynamics and the corresponding fate of the pharmaceutical residue Phenazone during artificial recharge of groundwater. Environ. Sci. Technol..

[39] Barkow IS, Oswald SE, Lensing H-J, Munz M (2021). Seasonal dynamics modifies fate of oxygen, nitrate, and organic micropollutants during bank filtration—Temperature-dependent reactive transport modeling of field data. Environ. Sci. Pollut. Res..

[40] Bloemendal M, Hartog N (2018). Analysis of the impact of storage conditions on the thermal recovery efficiency of low-temperature ATES systems. Geothermics.

[41] García-Gil A (2018). Occurrence of pharmaceuticals and personal care products in the urban aquifer of Zaragoza (Spain) and its relationship with intensive shallow geothermal energy exploitation. J. Hydrol..

[42] Labad F (2023). Occurrence, data-based modelling, and risk assessment of emerging contaminants in an alluvial aquifer polluted by river recharge. Environ. Pollut..

[43] Jurado A (2022). Occurrence of pharmaceuticals and risk assessment in urban groundwater. Adv. Geosci..

[44] Bunting SY (2021). Emerging organic compounds in European groundwater. Environ. Pollut..

[45] Escher BI, Fenner K (2011). Recent advances in environmental risk assessment of transformation products. Environ. Sci. Technol..

[46] Kottek M, Grieser J, Beck C, Rudolf B, Rubel F (2006). World Map of the Köppen-Geiger climate classification updated. Meteorol. Z..

[47] Kouzbour S, Stiriba Y, Gourich B, Vial C (2020). CFD simulation and analysis of reactive flow for dissolved manganese removal from drinking water by aeration process using an airlift reactor. J. Water Process Eng..

[48] Bagarinao T (1992). Sulfide as an environmental factor and toxicant: Tolerance and adaptations in aquatic organisms. Aquat. Toxicol..

[49] Barla M, Insana A (2023). Energy tunnels as an opportunity for sustainable development of urban areas. Tunn. Undergr. Space Technol..

[50] Becchio C (2017). Energy, economic and environmental modelling for supporting strategic local planning. Procedia Eng..

[51] Lee J-Y, Won J-H, Hahn J-S (2006). Evaluation of hydrogeologic conditions for groundwater heat pumps: analysis with data from national groundwater monitoring stations. Geosci. J..

[52] Casasso A, Sethi R (2019). Assessment and minimization of potential environmental impacts of ground source heat pump (GSHP) systems. Water.

[53] Li Z (2022). Influence of groundwater heat pump system operation on geological environment by hydro-thermal–mechanical–chemical numerical model. Appl. Therm. Eng..

[54] Kim, S.-K., Bae, G.-O. & Lee, K.-K. Numerical Analysis of Open-Loop and Closed-Loop Geothermal Heat Pump Systems. (2015).

[55] Koren K, Janža M (2019). Risk assessment for open loop geothermal systems, in relation to groundwater chemical composition (Ljubljana pilot area, Slovenia). Geologija.

[56] IDAEA-CSIC. *Avaluació de les possibilitats d’aprofitement geotèrmic a Can Batlló, districte de Sants-Montjuic, Barcelona*. 49 (2020).

[57] Wiese B (2011). Removal kinetics of organic compounds and sum parameters under field conditions for managed aquifer recharge. Water Res..

[58] Prommer H, Barry DA, Zheng C (2003). MODFLOW/MT3DMS-based reactive multicomponent transport modeling. Groundwater.

[59] Post VEA, Prommer H (2007). Multicomponent reactive transport simulation of the Elder problem: Effects of chemical reactions on salt plume development. Water Resour. Res..

[60] VDI. *VDI 4640 Blatt 2 - Thermische Nutzung des Untergrunds - Erdgekoppelte Wärmepumpenanlagen*. (2019).

[61] Diem S, Cirpka OA, Schirmer M (2013). Modeling the dynamics of oxygen consumption upon riverbank filtration by a stochastic–convective approach. J. Hydrol..

[62] Margot J, Maillard J, Rossi L, Barry DA, Holliger C (2013). Influence of treatment conditions on the oxidation of micropollutants by Trametes versicolor laccase. New Biotechnol..

[63] Sadef Y, Poulsen TG, Bester K (2014). Impact of compost process temperature on organic micro-pollutant degradation. Sci. Total Environ..

[64] Regnery J, Wing AD, Alidina M, Drewes JE (2015). Biotransformation of trace organic chemicals during groundwater recharge: How useful are first-order rate constants?. J. Contam. Hydrol..

[65] Henzler AF, Greskowiak J, Massmann G (2014). Modeling the fate of organic micropollutants during river bank filtration (Berlin, Germany). J. Contam. Hydrol..

[66] Kuehn W, Mueller U (2000). Riverbank filtration: An overview. J. AWWA.

[67] Scheytt TJ, Mersmann P, Heberer T (2006). Mobility of pharmaceuticals carbamazepine, diclofenac, ibuprofen, and propyphenazone in miscible-displacement experiments. J. Contam. Hydrol..

[68] Banzhaf S, Nödler K, Licha T, Krein A, Scheytt T (2012). Redox-sensitivity and mobility of selected pharmaceutical compounds in a low flow column experiment. Sci. Total Environ..

[69] Heberer T, Massmann G, Fanck B, Taute T, Dünnbier U (2008). Behaviour and redox sensitivity of antimicrobial residues during bank filtration. Chemosphere.

[70] Heberer T (2004). Field studies on the fate and transport of pharmaceutical residues in bank filtration. Groundw. Monit. Remediat..

[71] Rauch-Williams T, Hoppe-Jones C, Drewes JE (2010). The role of organic matter in the removal of emerging trace organic chemicals during managed aquifer recharge. Water Res..

[72] Banks, D. Thermogeological assessment of open-loop well-doublet schemes: a review and synthesis of analytical approaches. in *Proceedings of the International Association of Hydrogeologists (Irish Group) 27th Annual Groundwater Conference, ‘Groundwater: Opportunities and Pressures’* vol. 17 1149–1155 (2009).

[73] Wagner V, Bayer P, Bisch G, Kübert M, Blum P (2014). Hydraulic characterization of aquifers by thermal response testing: Validation by large-scale tank and field experiments. Water Resour. Res..

[74] Marzan I, Fernandez M, Puig C, Berastegui X, Serra L (2011). Geothermal Atlas of Catalonia V.0.

[75] Zurmuhl DP (2019). Hybrid geothermal heat pumps for cooling telecommunications data centers. Energy Build..

[76] AEMET. Valores extremos absolutos - Selector - Agencia Estatal de Meteorología - AEMET. Gobierno de España. http://www.aemet.es/es/serviciosclimaticos/datosclimatologicos/efemerides_extremos* (2022).

