# Author Correction: Potential of low-enthalpy geothermal energy to degrade organic contaminants of emerging concern in urban groundwater

**DOI:** 10.1038/s41598-023-33394-7

**Published:** 2023-04-14

**Authors:** Estanislao Pujades, Anna Jurado, Laura Scheiber, Marc Teixidó, Rotman A. Criollo Manjarrez, Enric Vázquez-Suñé, Victor Vilarrasa

**Affiliations:** 1 Department of Geosciences, Institute of Environmental Assessment and Water Research (IDAEA), Severo Ochoa Excellence Center of the Spanish Council for Scientific Research (CSIC), Jordi Girona 18-26, 08034 Barcelona, Spain; 2 Global Change Research Group (GCRG), IMEDEA, CSIC-UIB, Miquel Marqués 21, 07190 Esporles, Spain

Correction to: *Scientific Reports* 10.1038/s41598-023-29701-x, published online 14 February 2023

The Supplementary Information published with this Article contained an error.

In “Appendix C”, Figures C1 and C2, the label of the Y-axis “Concentration (ng/L)” was incorrectly given as “Concentration mg/l”.

This error has now been corrected in the Supplementary Information file that accompanies the original Article.

## Supplementary Information

Supplementary Information.

