# Supplementary material for: Potential of low-enthalpy geothermal energy to degrade organic contaminants of emerging concern in urban groundwater
Source: Sci Rep. 2023 Feb 14;13:2642. doi: 10.1038/s41598-023-29701-x (PMC9929318; doi:10.1038/s41598-023-29701-x)
Supplement: Supplementary file 1 — Supplementary Information. [file 41598_2023_29701_MOESM1_ESM.pdf]

**Supplementary material for:**

# Potential of low-enthalpy geothermal energy to degrade organic contaminants of emerging concern in urban groundwater

Estanislao Pujades[1,*], Anna Jurado[1], Laura Scheiber[1], Marc Teixidó[1], Rotman A. Criollo Manjarrez[2], Enric Vázquez-Suñé[1], Victor Vilarrasa[2]

[1] Department of Geosciences, Institute of Environmental Assessment and Water Research (IDAEA), Severo Ochoa Excellence Center of the Spanish Council for Scientific Research (CSIC), Jordi Girona 18–26, 08034 Barcelona, Spain.

[2] Global Change Research Group (GCRG), IMEDEA, CSIC-UIB, Miquel Marquès, 21, 07190 Esporles, Spain

## Appendix A

### *A1. Synthetic approach*

A1.1. General description

The synthetic models consist in a groundwater heat pump (GWHP) system located in a fully-saturated sandy aquifer with a thickness of 20 m. It is assumed that the aquifer is homogeneous and it is located just below the water table. The synthetic models are used to study the evolution of carbamazepine in an aquifer affected by a GWHP facility. This substance is selected because is frequently detected in urban areas [1], showing a recalcitrant behaviour [2]. It is considered that groundwater has $1.1 \cdot 10^{-10}$ mol $L^{-1}$ of carbamazepine. The aquifer properties and parameters are summarized in Table A1.

Groundwater flows from West to East boundaries with a natural hydraulic gradient (*i*) of 0.005. The aquifer length (West to East boundaries) and width (South to North boundaries) are 1,000 and 500 m, respectively (Figure A1).

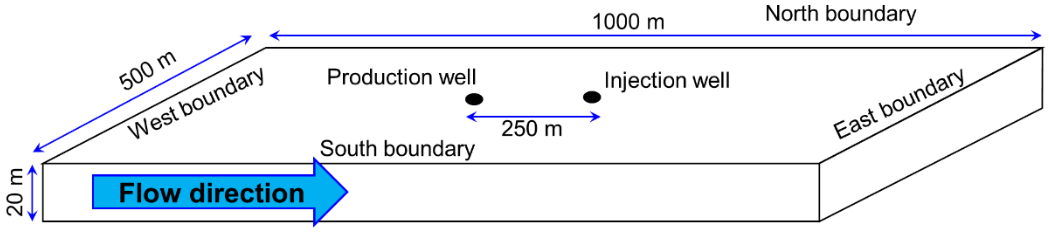


Figure A1. Schematic description of the synthetic case.

The GWHP system consists in an open loop scheme. The pumping well (i.e., production well) is placed upgradient while the re-injection well is located downgradient (Figure A1). Pumping and injection rates (*Q*) do not vary over time and are equal to 432 $m^3$ $d^{-1}$ (i.e., 5 l $s^{-1}$). To ensure that thermal breakthrough ($t_{BR}$) does not occur, the distance between the two wells, 250 m, is calculated according to Eq. (4) of the main text.

Table A1. Aquifer parameters used in the synthetic model. Values agree with those typical for sandy aquifers (*K*: Domenico and Schwartz, 1998; *θ*: Woessner and Poeter, 2020;$D_L$: Schulze-Makuch, 2005). $D_T$ is chosen ten time lower than $D_L$, which is common practice [6] and $D_m$ is selected in agreement with the reference values specified in the guidelines for thermal use of the underground of the German Engineer Association [7].

| Parameter | Value |
|---|---|
| Hydraulic conductivity (*K*) | 10 $md^{-1}$ |
| Anisotropy factor | 1 |
| Effective porosity ($\theta_{eff}$) | 0.05 |
| Longitudinal Dispersion ($D_L$) | 10 m |
| Transversal Dispersion ($D_T$) | 1 m |
| Thermal diffusivity ($D_m$) | $1.86 \cdot 10^{-6}$ $m^2d^{-1}$ |
| Boundary conductance | 0.2 $md^{-1}$ |

A1.2. Numerical approach

PHT3D [8] is the code used to develop the numerical model. The total simulated time is 10 years and chosen time steps are of 10 and 1 days for the flow and transport problem, respectively. The mesh consists in one layer that is divided in 5,000 equally-sized squares with an area of 100 $m^2$.

A third type boundary condition (BC) is applied in the upgradient (West) and downgradient (East) boundaries to impose a hydraulic gradient of 0.005. This BC allows reducing the size of the model by assuming that the head is unperturbed at a given distance from the boundary. It is considered that the influence of the GWHP facility is negligible 2,000 m far away from the current boundaries. Then, it is assumed that the head does not vary at a distance of 2,500 m from the GWHP facility. The parameter defining the conductance of the boundary is computed

considering this distance (Table A1). Finally, the flow is prescribed in the production and injection wells.
Concerning the transport BCs, the concentration is prescribed at the upgradient (i.e., West) boundary ($1.1{\cdot}10^{-10}$ mol $L^{-1}$) and at the injection well (the concentration is equal to that of the pumped water in the production well). In addition, a constant input of mass of $1.1{\cdot}10^{-10}$ mol $d^{-1}$ $m^2$ is implemented. The value of the input mass has been chosen to reach initial concentrations near to $1.1{\cdot}10^{-10}$ mol $L^{-1}$ of carbamazepine in the whole domain. Initial conditions (piezometric head and concentration) are calculated by simulating a period of 10 years without considering the presence of the GWHP facility. The behavior of carbamazepine (CBZ) is modeled using Monod kinetics, the Eq. (1) and (2) and the parameters defined in Table 2 of the main text.

A1.3. GWHP scenarios
Two different GWHP scenarios (Sch1 and Sch2) are simulated with the synthetic model to evaluate how the use of the GWHP facility affects the behaviour of the selected OCEC. The temperature of injected water is computed according with Eq. (6) of the main text. Scenario Sch1 considers that the GWHP facility is only used for cooling and a constant $P_{GW}$ is obtained. As a result, the temperature of the injected water (35ºC) is also constant. Assuming a constant groundwater temperature of 20ºC under natural conditions and considering Eq. (6) of the main text, the $P_{GW}$ is 313,800 W (i.e., 225,936 kWh per month). In scenario Sch 2, $P_{GW}$ is computed by considering a hypothetical 5-storey hospital located in Barcelona (Spain). The energy requirements of this hypothetical hospital are computed by using the software CLIMA [9]. This software, which has been developed by the Polytechnic University of Valencia, is freely distributed by the Spanish Technical Association for Air Conditioning and Refrigeration (ATECYR). The cooling and heating demands of such hospital are in the software CLIMA. Figure A2 shows the energy requirements for the simulated building during a year and the expected temperature for the injected water (blue line) computed by Eq. (6). The same requirements are assumed for all the simulated years.

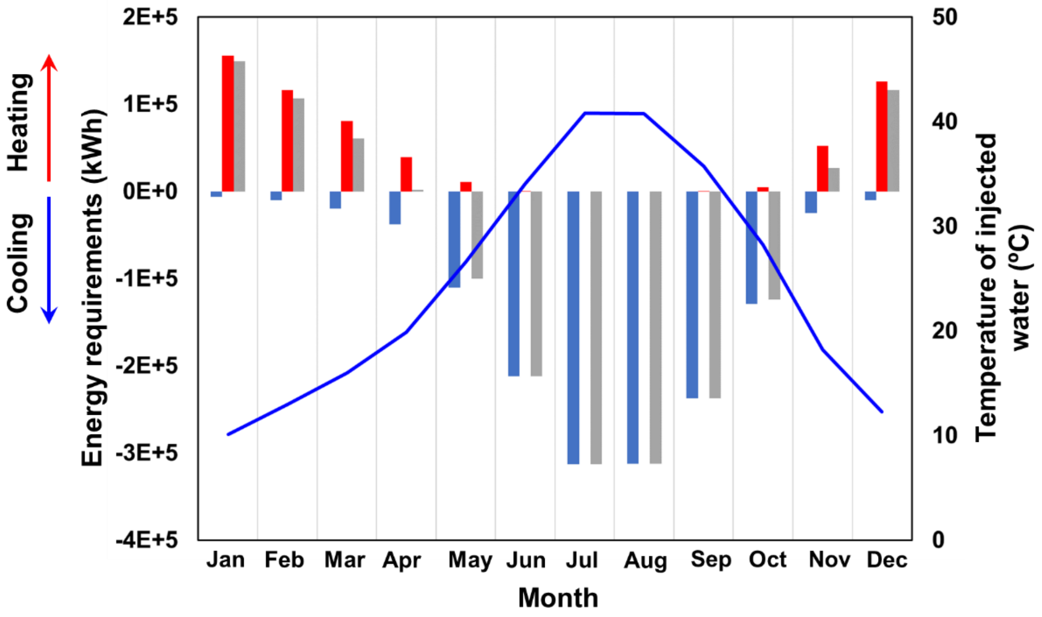


*Figure A2. Energy required for the climatization of a hospital along a year as simulated by CLIMA [9]. Columns refer to energy needed for cooling (blue), heating (red) and the net energy (grey). Positive values refer to energy needed for heating while negative ones refer to energy required for cooling. The continuous blue line indicates the temperature of the injected water computed by using Eq. (6) to meet the energy requirements of climatization for scenario Sch2.*

## A1.4. Results

### *A1.4.1. Concentration and groundwater temperature across the aquifer*

Injection temperature in scenario Sch1 is constant (35 ºC) and creates a plume of hot groundwater from near the production well to the downgradient boundary where the groundwater temperature reaches 34.7ºC (Figure A3a). Not only does hot groundwater flow downgradient following the hydraulic gradient of the aquifer, but also upgradient dragged by the production well. Nonetheless, no thermal breakthrough occurs, as expected, because the system has been designed to avoid it through Eq. (4). The degradation rate of carbamazepine increases with temperature according to Eq. (1), decreasing its concentration within the thermal plume (Figure A3b). In Figure A3b, the concentration of carbamazepine ($C_{CBZ}$) is normalized by the concentration under unperturbed (i.e., initial) conditions ($C_{CBZ,0}$). The concentration presents a local maximum around the injection well because the degradation of carbamazepine is not instantaneous and requires a certain time to lower the concentration as water flows from the injection well into the aquifer. The minimum normalized concentration is about 0.15, which means

that the concentration of carbamazepine decreases up to the 85 % in comparison with the concentration under unperturbed conditions.

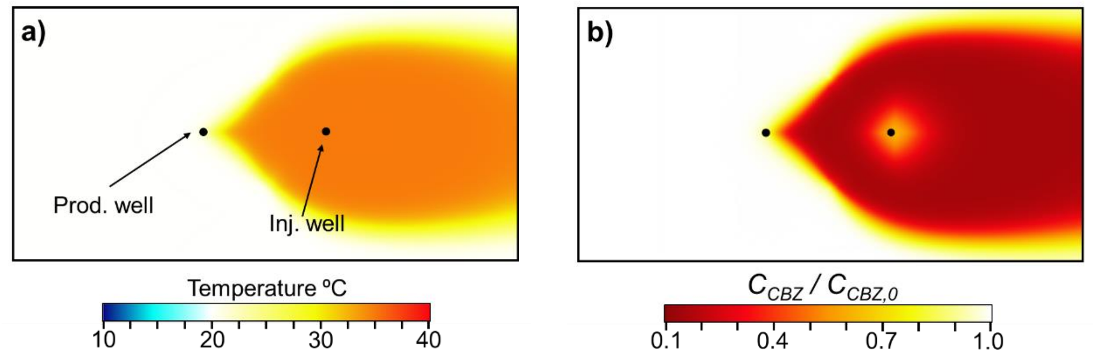


*Figure A3. (a) Temperature distribution and (b) normalized concentration of carbamazepine* ($C_{CBZ}$) by the concentration under unperturbed conditions ($C_{CBZ.0}$) *across the simulated aquifer after 10 years of operation for scenario Sch1 (water is injected at a constant temperature of 35ºC).*

In scenario Sch2, the temperature of the injected water varies depending on the climatization requirements along the year (Figures A4a and A4b correspond to the hottest month, August, and Figures A4c and A4d to the coldest month, January). Thus, injected water is hotter (up to 40 ºC) than the aquifer temperature (20 ºC) when GWHP is used for cooling and colder (down to 10 ºC) when it is used for heating. As a result, the temperature around the injection well oscillates (Figures A4a and A4c). Despite the groundwater temperature oscillates at the injection point along the year, a thermal plume with higher temperature than that under unperturbed conditions is generated from near the production well to the downgradient boundary. The temperature increase is due to an imbalance between heating and cooling demands, being higher the latter (Figure A2). The maximum temperature reached on the downgradient boundary is around 25ºC, being lower than that in Sce1. Thus, the overall carbamazepine attenuation is lower than in scenario Sch1 (Figures A4b and A4d).

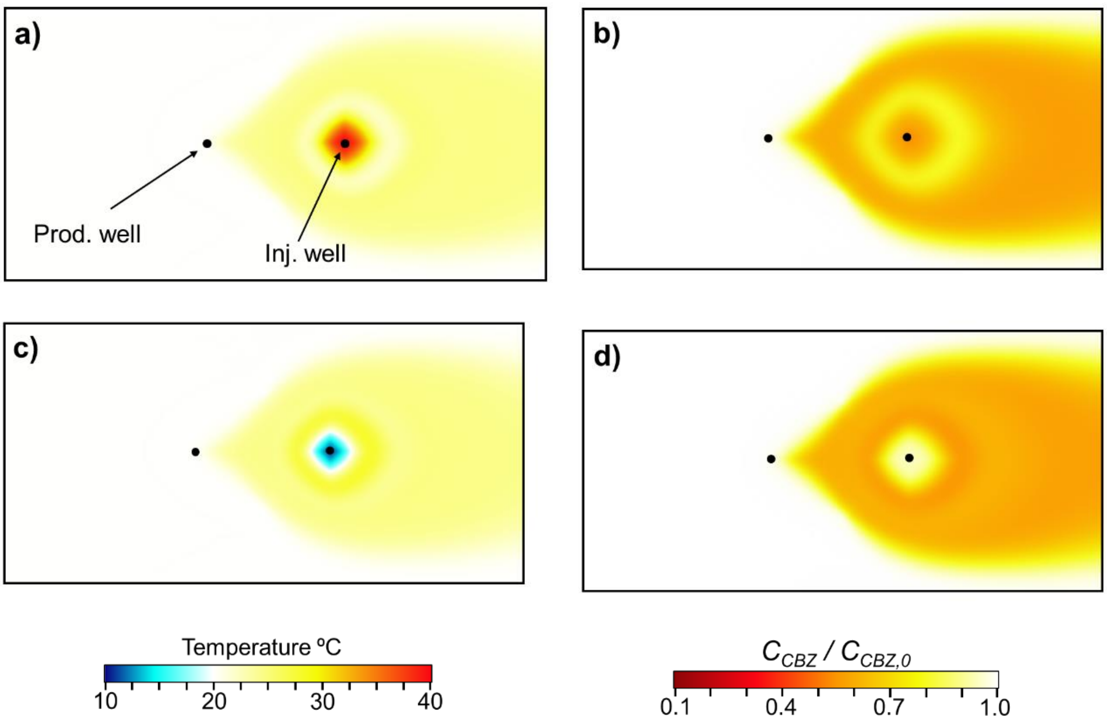


*Figure A4. Temperature distribution for (a) August and (c) January and normalized concentration of carbamazepine for (b) August and (d) January across the simulated aquifer for scenario Sch2. Results are shown for the months when the temperature and concentration reach their maximum (a and b) and minimum values (c and d) (i.e., August and January, respectively).*

Once the thermal plume is created and a quasi-steady state is reached, the concentration of carbamazepine only varies around the injection well, which is the only area where groundwater temperature fluctuates along the year (Figure A4). As expected, the minimum concentration of carbamazepine around the well is observed when the groundwater temperature is high and vice versa (Figures A4b and A4d). In the rest of the aquifer, the degradation of carbamazepine increases and its concentration is lower than that under unperturbed conditions (Figures A4b and A4d). Note that when the temperature of the injected water is the maximum, there is a ring-shaped area around the injection well with higher concentration than in the rest of the area affected by the facility (Figure A4a). This local increase is related to the period in which the injected water is cold and the degradation velocity decreases. During this phase, a local maximum of carbamazepine occurs at the injection well (Figure A4d). As injection temperature progressively increases, the cold groundwater with high concentration of carbamazepine and low degradation capacity is displaced away from the injection

well, generating the ring-shaped area. The concentration of carbamazepine in this area progressively decreases, as it is heated up and mixed with hot water with low concentration of carbamazepine. The minimum concentration of carbamazepine reached in scenario Sch2 is about 43% lower than the concentration under unperturbed conditions, and thus, 42% higher than the minimum concentration in scenario Sch1. Thus, the degradation velocity is higher than under unperturbed conditions, but lower than in scenario Sch1 because the alternating demand for heating and cooling leads to a lower temperature increase.

*A1.4.2. Evolution of concentration and groundwater temperature on the downgradient boundary*

Figure A5a shows the evolution of the concentration of carbamazepine normalized by the initial concentration (i.e., under unperturbed conditions) on the downgradient boundary. The computed concentration is the average concentration along the downgradient boundary for the simulated GWHP scenarios, Sch1 (red line) and Sch2 (blue line). Figure A5b shows the groundwater temperature at the downgradient boundary. The temperature is calculated by considering the whole volume of water crossing the boundary. The concentration (Figure A5a) during the first year and a half is equal than that under unperturbed conditions, meaning that the influence of the GWHP facility has not reached the downgradient boundary yet. After one year and a half, the concentration in scenario Sch1 starts to decrease. A steady state is not reached during the simulated period, indicating that concentrations of carbamazepine can reach lower values in longer operation periods. The normalized concentration after ten years decays up to 0.36 as a result of the increasing groundwater temperature in scenario Sch1. In scenario Sch2, the concentration decreases more slowly than in Sch1 because the alternating hot and cold water injection leads to a lower temperature increase. A steady state is neither reached during the simulation time, and the normalized concentration at the end of the simulation decreases to 0.7. Concentration evolution correlates with the temperature of the groundwater in the downgradient boundary (Figure A5b). In both GWHP scenarios, the temperature progressively increases, but a steady state is not reached. The temperature of the groundwater crossing the downgradient boundary increases up to 30 and 23 ºC for Sch1 and Sch2 scenarios,

respectively. As a result, more carbamazepine is expected to be degraded in scenario Sch1, which supports the results shown in Figure A5a. Variations of the concentration of carbamazepine in the groundwater crossing the downgradient boundary are observed faster than that of temperature. This fact is related to the retardation factor, which is higher for heat than for carbamazepine.

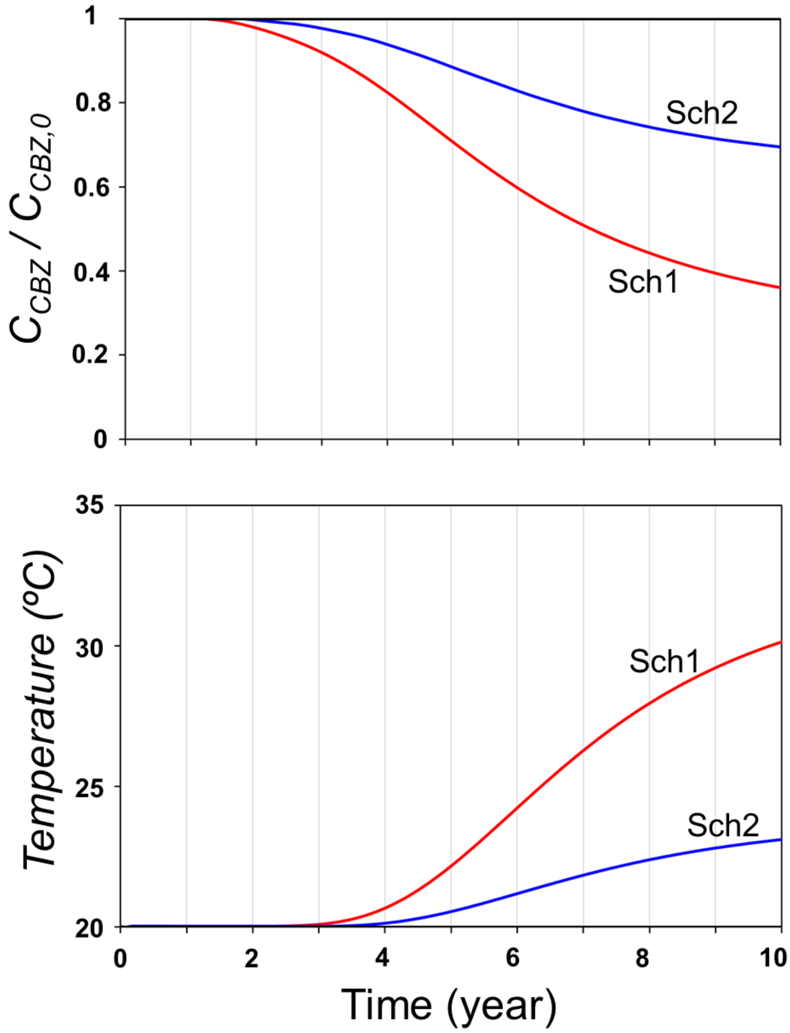


*Figure A5. a) Normalized concentration of carbamazepine in the groundwater that flows-out the model through the downgradient boundary. The concentration of carbamazepine ($C_{Carb}C_{CBZ}$) is normalized by the concentration under unperturbed conditions ($C_{Carb\text{-}INI}C_{CBZ,0}$). b) Temperature of groundwater that flows-out the model through the downgradient boundary. Temperature is computed considering all water crossing the boundary.*

**Appendix B**

*B1. Sensitivity analysis*

This appendix shows the results of the real-based model varying the hydraulic conductivity (*K*), the effective porosity $\theta_{eff}$, the longitudinal and transversal dispersivities ($D_L$ *and* $D_T$) and the thermal diffusivity ($D_m$). The simulations are developed considering scenario Sce4. This scenario has been selected because it is the most favourable to increase the degradation capacity of the aquifer against diclofenac and carbamazepine.

*B1.1. Hydraulic conductivity*

The sensitivity of the model to *K* is assessed by increasing and decreasing its value one order of magnitude with respect to that measured in the field (3.3 m $d^{-1}$) and comparing the results with those of the original scenario (Sce4). Compared results are the distribution of the normalized concentrations of diclofenac (Fig. B1a) and carbamazepine (Fig. B1b) and the evolution of the normalized concentration of diclofenac (Fig. B2a) and carbamazepine (Fig. B2b) at the downgradient boundary.

The lowest concentrations occur when *K* is reduced by one order of magnitude (i.e., 0.33 m $d^{-1}$) (Fig. B1). In addition, the area where the concertation of both OCECs is reduced is also enlarged when reducing *K*. Similar observations can be drawn from Figure B2. The higher the hydraulic conductivity, the faster the decrease in the concentration at the output boundary. However, the tendency indicates that in the long term, the lowest concentration of OCECs is reached by reducing *K.* Despite the fast initial concentration decrease with the higher *K*, the normalized concentration stabilizes after 5 years of operation with values of around 0.8 and 0.6 for diclofenac and carbamazepine, respectively. However, when *K* is reduced one order of magnitude (i.e., 0.33 m $d^{-1}$) the effect of the GWHP facility is not observed during the first two years (there is only a small decrease as a result of the starting of the pumping and injection activities). Later, the concentrations decrease slowly in comparison with the other scenarios, but at the end of the simulated period, the tendency indicates that the steady state is far from being reached and the concentration will continue decreasing until reaching lower concentrations than for the other two scenarios.

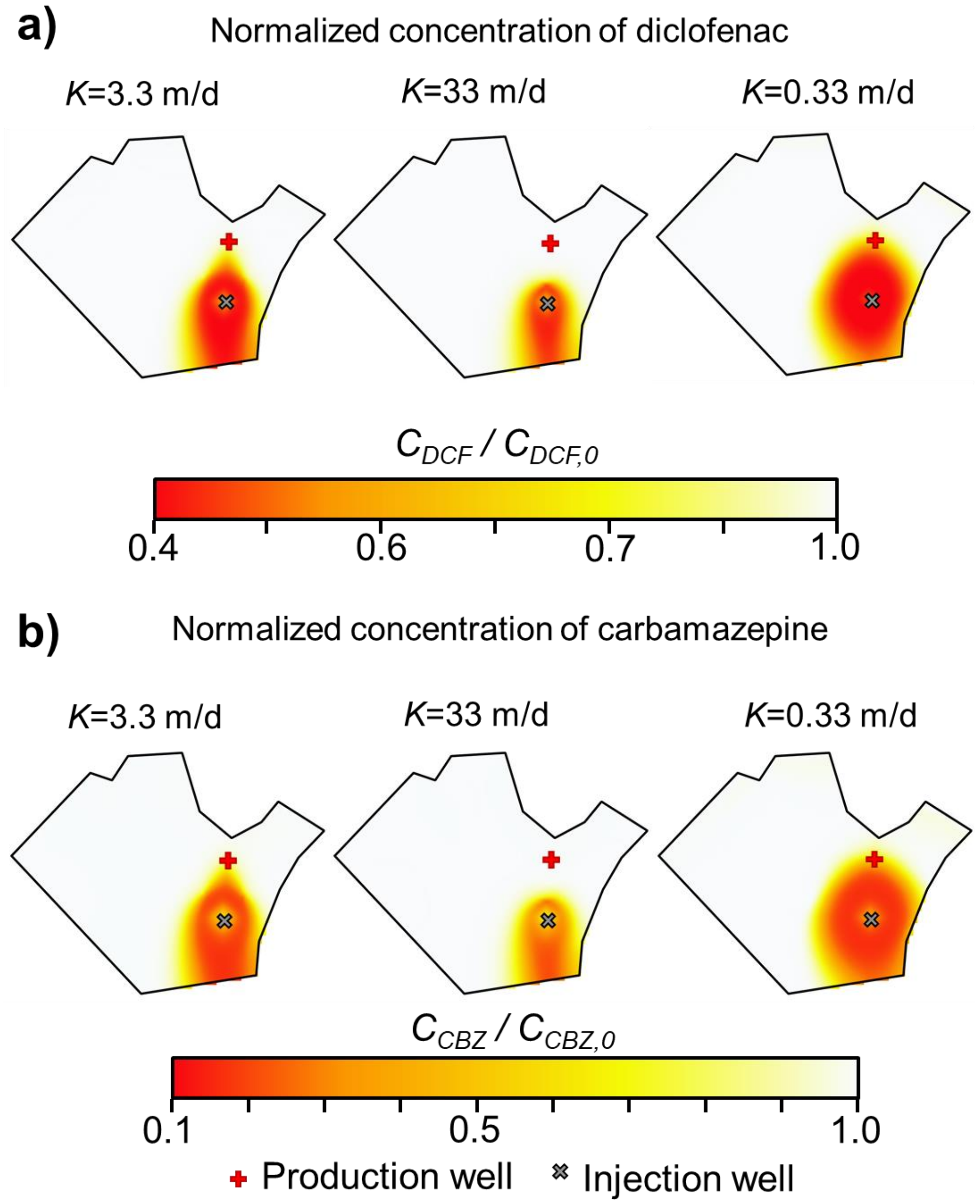


*Figure B1. Normalized concentration of diclofenac (a – $C_{DCF}/C_{DCF,0}$) and carbamazepine (b - $C_{CBZ}/C_{CBZ,0}$) after 10 years of operation for scenario Sce4 of the real-based model. The hydraulic conductivity has been decreased (0.33 m $d^{-1}$) and increased (33 m $d^{-1}$) one order of magnitude with respect to that measured in the field (3.3 m $d^{-1}$). These results highlight the importance of developing a detailed hydrogeological characterization.*

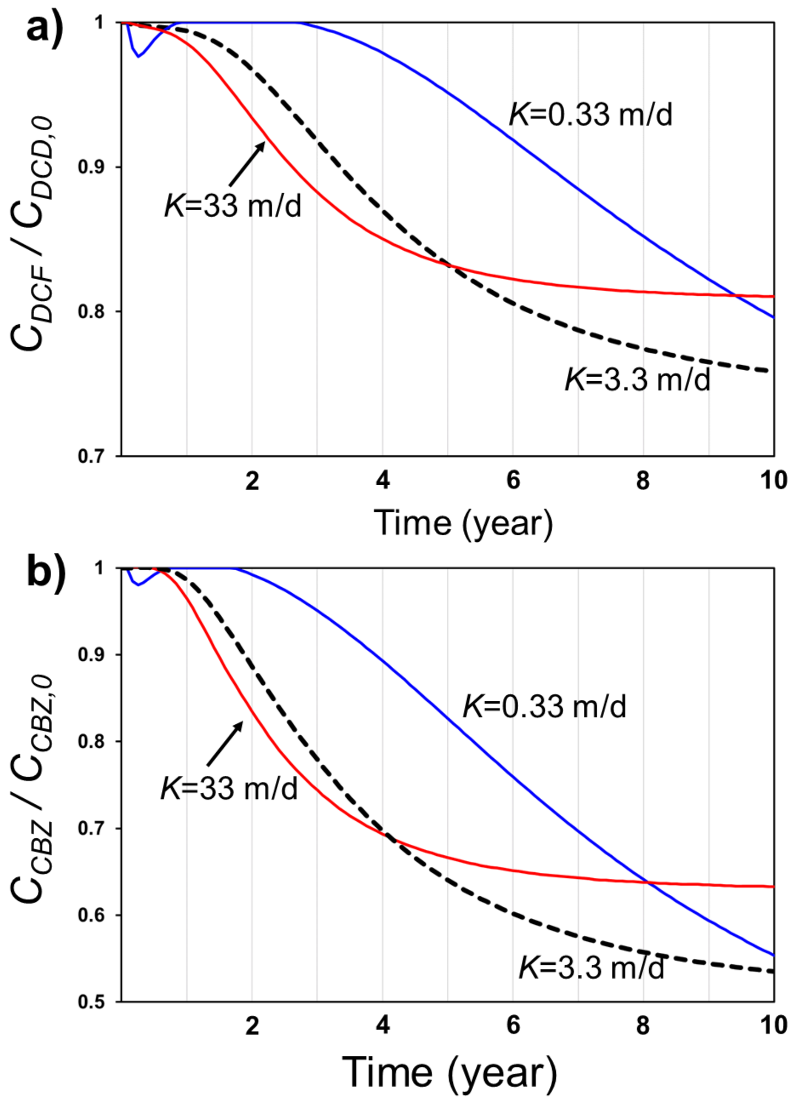


*Figure B2. Normalized concentration of diclofenac (a – $C_{DCF}/C_{DCF,0}$) and carbamazepine (b - $C_{CBZ}/C_{CBZ,0}$) at the groundwater flowing out of the model through the downgradient boundary. The normalized concentration is computed by integrating the concentration of all water that crosses the boundary and considering the concentration under unperturbed conditions.*

*B1.2.* Porosity, the longitudinal and transversal dispersivities and the thermal diffusivity

The sensitivity of the model to $\theta_{eff}$, $D_L$, $D_T$ *and* $D_m$ is assessed by comparing the results of the original simulation (Sce4) with those obtained when their values are modified. In total, three scenarios are simulated. At the first scenario (SceB1), $\theta_{eff}$, is reduced one order of magnitude. $D_L$, $D_T$ are also reduced one order of magnitude in the second scenario (SceB2). Finally, $D_m$ is reduced two orders of magnitude in the third scenario (SceB3).

The influence of the assessed parameters in the concentration evolution of both compounds is similar (Fig. B3). The concentration decreases faster when $\theta_{eff}$ is reduced, but at the end of the simulation its value is the same than in the original scenario. This behaviour is due to the fact that groundwater velocity increase when reducing $\theta_{eff}$ and, thus, the effects of the GWHP facility reach faster the downgradient boundary. The reduction of the dispersivities $D_L$, $D_T$ slightly delays the concentration reduction of both compounds on the downgradient boundary. Finally, the concentration evolution on the downgradient boundary of the studied OCECs is insensitive to the thermal diffusivity.

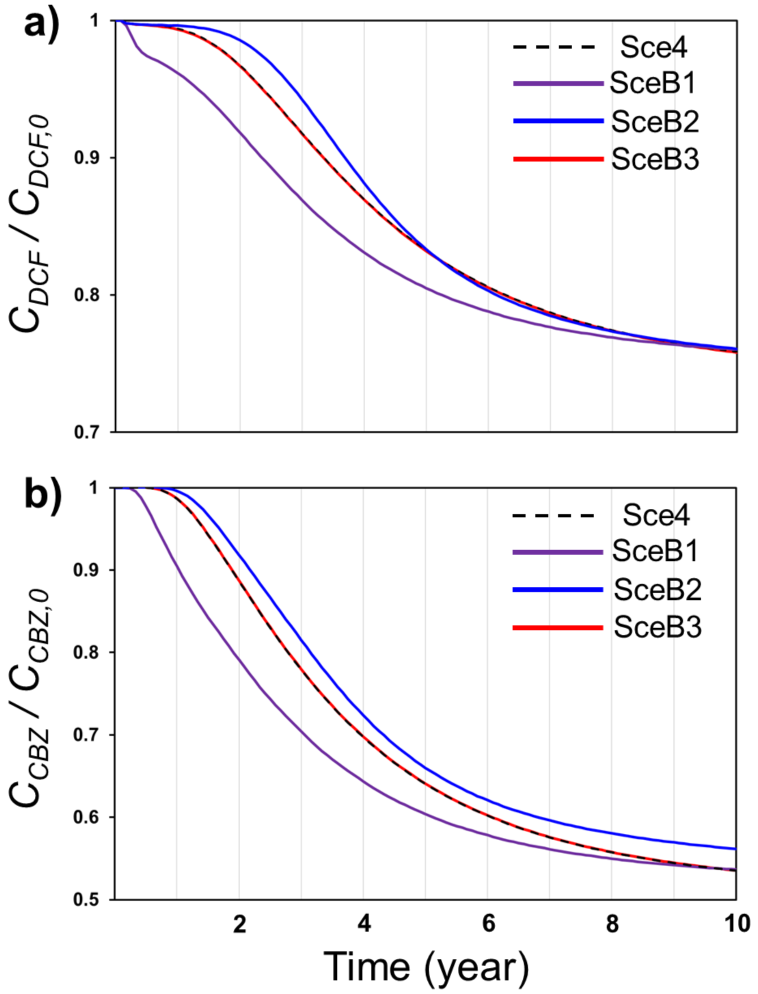


*Figure B3. Normalized concentration of diclofenac (a – $C_{DCF}/C_{DCF,0}$) and carbamazepine (b - $C_{CBZ}/C_{CBZ,0}$) at the groundwater flowing through the downgradient boundary for scenarios Sce4, SceB1, SceB2 and SceB3. The normalized concentration is computed by integrating the concentration of all water that crosses the boundary and dividing it by the concentration under unperturbed conditions. The results are obtained by varying the values of $\theta_{eff}$ (SceB1), $D_L$, $D_T$ (SceB2) and $D_m$ (SceB3) with respect the original scenario (Sce4).*

**Appendix C**

Concentration of OCECs reported at the study site is shown in the two plots of Figure C1.

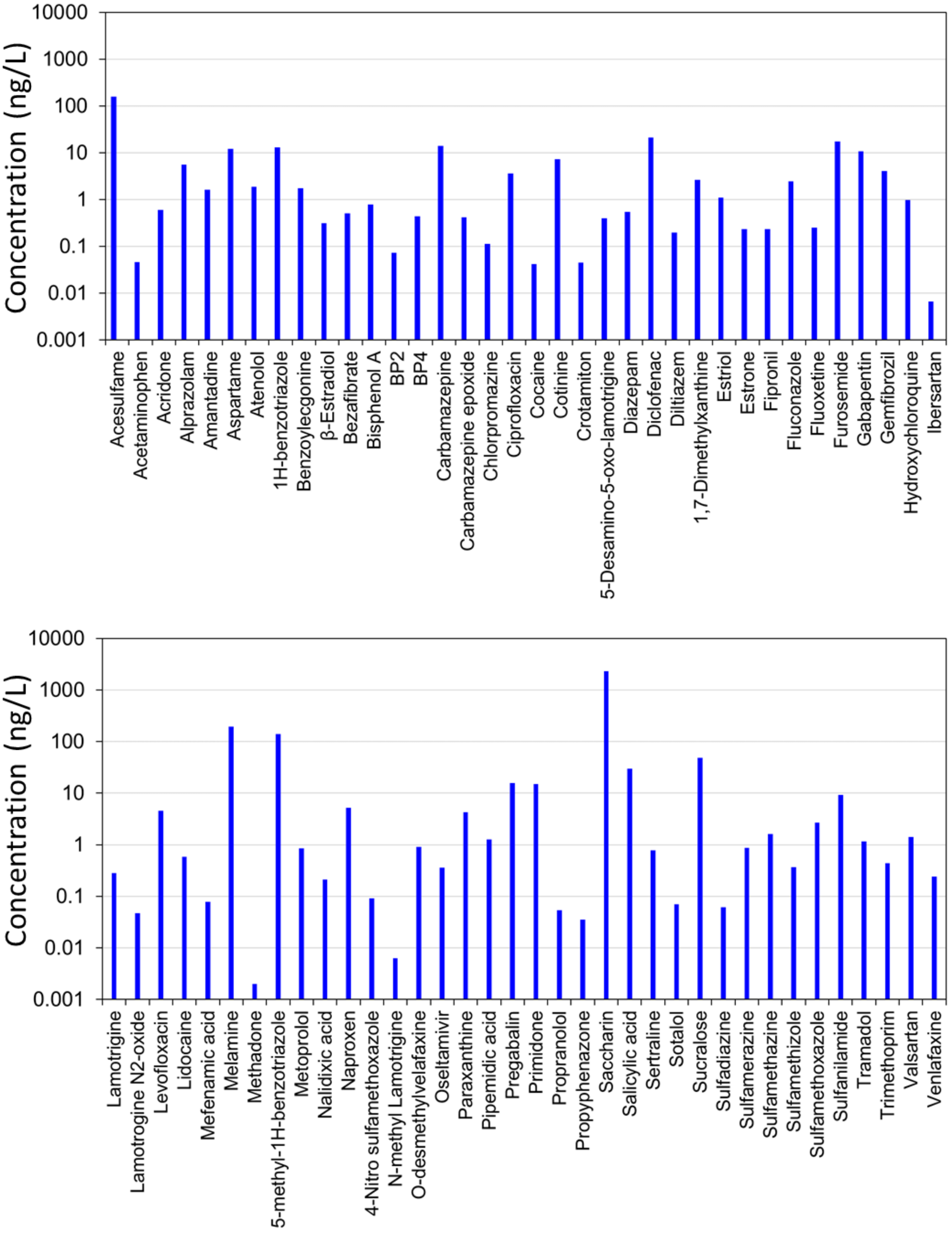


Figure C1. Concentration of OCECs reported at the study site

## References


1. Jurado, A. *et al.* Occurrence of carbamazepine and five metabolites in an urban aquifer. *Chemosphere* **115**, 47–53 (2014).
2. Clara, M., Strenn, B. & Kreuzinger, N. Carbamazepine as a possible anthropogenic marker in the aquatic environment: investigations on the behaviour of Carbamazepine in wastewater treatment and during groundwater infiltration. *Water Research* **38**, 947–954 (2004).
3. Domenico, P. A. & Schwartz, F. W. *Physical and Chemical Hydrogeology*. (Wiley, 1998).
4. Woessner, W. & Poeter, E. *Hydrogeologic Properties of Earth Materials and Principles of Groundwater Flow*. (2020).
5. Schulze-Makuch, D. Longitudinal dispersivity data and implications for scaling behavior. *Groundwater* **43**, 443–456 (2005).
6. Zech, A. *et al.* A Critical Analysis of Transverse Dispersivity Field Data. *Groundwater* **57**, 632–639 (2019).
7. VDI. *VDI 4640 Blatt 2 - Thermische Nutzung des Untergrunds - Erdgekoppelte Wärmepumpenanlagen*. (2019).
8. Prommer, H., Barry, D. A. & Zheng, C. MODFLOW/MT3DMS-Based Reactive Multicomponent Transport Modeling. *Groundwater* **41**, 247–257 (2003).
9. Pinazo, J. M., Soto, V. & Sarabia, E. CLIMA. (2019).
